# Polymer-based biopotential electrode-leadwire prototype for ECG sensing during X-ray and CT examinations

**DOI:** 10.3389/fbioe.2026.1880492

**Published:** 2026-09-30

**Authors:** Marek Penhaker, Jan Polomik, Jan Kijonka, Jaroslav Stejskal, Martin Cerny, Dominik Vilimek, Peter Kneppo, Ladislav Janousek, Ewaryst Tkacz, Jan Kubicek

**Affiliations:** 1 Faculty of Electrical Engineering and Computer Science, VSB - Technical University of Ostrava, Ostrava Poruba, Czechia; 2 Faculty of Electrical Engineering, University of Zilina, Zilina, Slovakia; 3 Institute of Macromolecular Chemistry, Academy of Sciences of the Czech Republic, Prague, Czechia; 4 Department of Biomedical Technology, Faculty of Biomedical Engineering, Czech Technical University in Prague, Kladno, Czechia; 5 Academy of Silesia, Prof. Zbigniew Religa Faculty of Medical Sciences, Department of Clinical Engineering, Katowice, Poland

**Keywords:** biopotential electrode, CT, ECG, leadwire, polyaniline, polymer, polypyrrole, x-ray

## Abstract

**Introduction:**

Continuous ECG monitoring during X-ray and CT examinations is crucial for critically ill patients; however, standard Ag/AgCl electrodes and metallic leadwires generate image artifacts that may compromise diagnostic quality. This study aimed to develop and evaluate a non-metallic, radiolucent polymer-based ECG electrode–leadwire system enabling artifact-free ECG sensing during radiological imaging.

**Materials and Methods:**

Biocompatible conductive polymers, polypyrrole (PPy) and polyaniline (PANI), were polymerized *in situ* onto cotton and carbon substrates to fabricate dry textile electrodes and carbon-fiber leadwires. The polymer-based electrode-leadwire prototype was compared to standard Ag/AgCl electrodes and metallic leadwires in terms of ECG signal quality and radiolucency. Radiolucency was evaluated using CT and X-ray imaging on phantom models and a veterinary subject, with CT conspicuity quantified by Hounsfield unit attenuation difference |ΔHU|. ECG signal quality was assessed using complementary time-domain metrics, including Pearson correlation, root-mean-square error (RMSE), mean absolute error (MAE), signal-to-noise ratio (SNR), and baseline variability, together with the frequency-domain metric magnitude-squared coherence (MSC), under static and simulated CT motion conditions.

**Results:**

PPy-coated cotton electrodes demonstrated high agreement with Ag/AgCl references with Pearson correlation coefficients ranging from 0.93 to 0.99 and magnitude-squared coherence values ranging from 0.81 to 0.99 across all tested configurations. Relative R-wave amplitude deviations remained below 13%. PANI-coated carbon-fiber leadwires exhibited low resistance (≈11 Ω/m) and stable signal transmission. CT and X-ray imaging showed complete radiolucency of conductive polymer components, with only faint visibility of auxiliary non-conductive materials under high-contrast CT settings. Simulated CT table motion introduced only minor signal degradation with RMSE values ≤0.05 mV and preserved signal-to-noise ratio and baseline stability within ranges commonly considered acceptable for clinical ECG monitoring.

**Conclusion:**

The proposed dry, non-metallic polymer-based ECG prototype provides artifact-free ECG monitoring during X-ray and CT imaging while preserving clinically relevant signal quality. The results demonstrate the feasibility of uninterrupted ECG monitoring during radiological examinations without introducing image artifacts associated with conventional metallic ECG systems.

## Graphical Abstract

**Figure:**
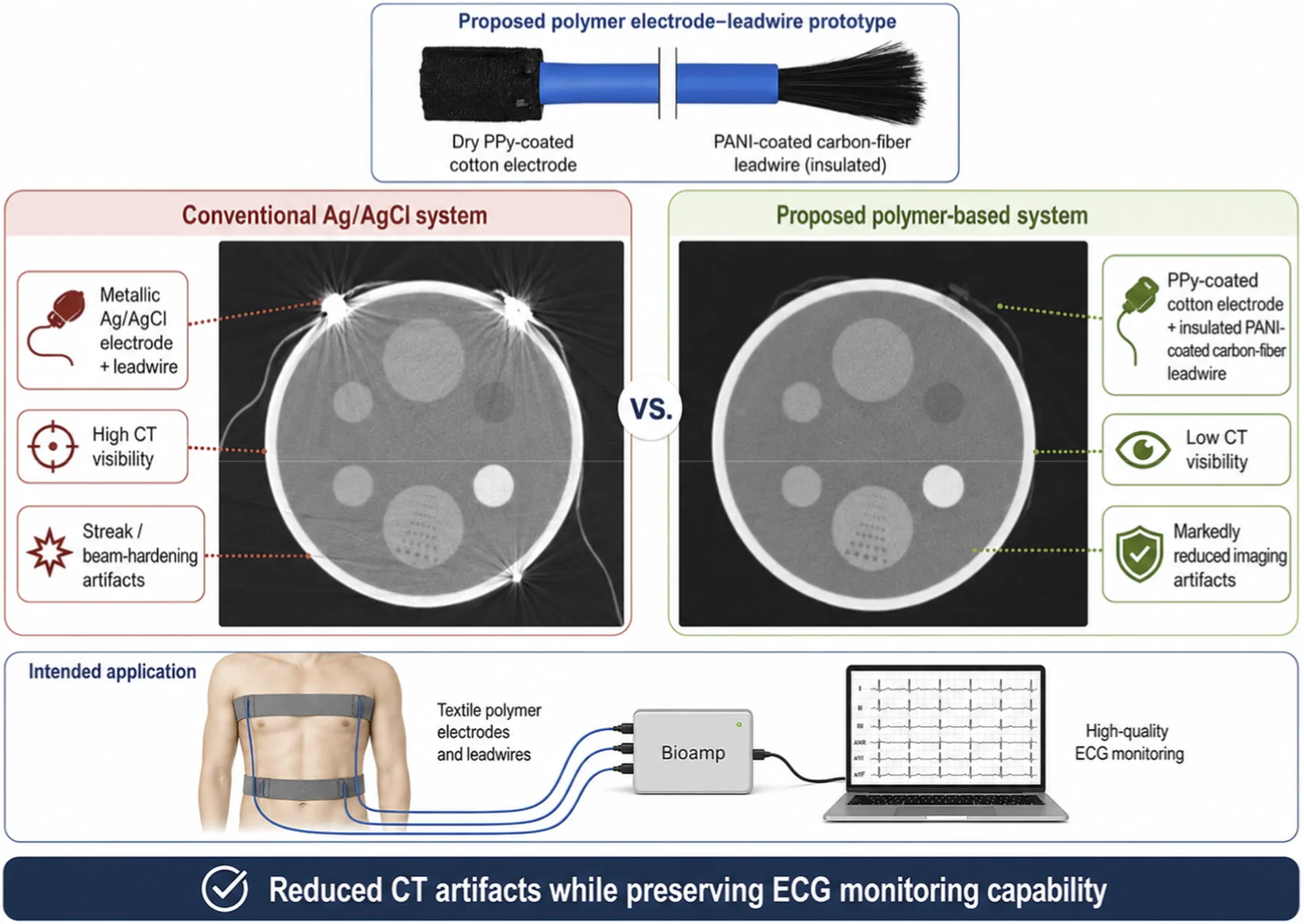

## 1 Introduction

In patients at risk of heart failure (e.g., trauma patients), continuous cardiac activity sensing is necessary even during X-ray and computed tomography (CT) examinations, where any interruption for just a few minutes can be risky and life-threatening. However, in current clinical practice this is often not feasible, because standard metal-based Ag/AgCl electrodes are unsuitable for electrocardiographic (ECG) monitoring during X-ray and CT due to severe image artifacts. These artifacts arise when high atomic number (high-Z) materials are present in the scanned region (Zègre-Hemsey et al., 2016; Sampson, 2018; Harjola et al., 2018; Bekmukhametov et al., 2016).

In diagnostic X-ray imaging, photons are attenuated mainly through three interactions: the photoelectric effect, Compton scattering, and higher energies, pair production. For photon energies typical of diagnostic radiology (20–120 keV), the photoelectric effect dominates, and its probability increases rapidly with atomic number *Z* and photon energy *E*, commonly approximated as proportional to *Z* ^5^/*E* ^3^ (Cierniak, 2011; Barrett and Keat, 2004). Materials containing silver (*Z* = 47), such as metals used in Ag/AgCl electrodes, therefore absorb low-energy photons far more strongly than soft tissues (average *Z* ≈ 7), producing bright streaks and dark shadows in reconstructed CT images (Cierniak, 2011). After photoelectric absorption, ejected photoelectrons and secondary Auger electrons release additional localized energy, amplifying the artifact intensity. In contrast, polymeric or carbon-based materials with low atomic numbers (*Z* ≤ 6) exhibit negligible photoelectric absorption and do not introduce visible image distortions (Katsura et al., 2018).

Modern CT correction algorithms are optimized for soft-tissue and bone attenuation but may remain ineffective when metallic components are present, which explains why even small Ag- or Ti-based electrode parts can cause streaking artifacts. Intrinsically conductive polymers, including polypyrrole (PPy), polyaniline (PANI), and PEDOT:PSS, have attracted considerable interest in bioelectronics because of their electrical conductivity, mechanical compliance, low weight, and ability to form conformal interfaces with biological tissues. Recent reviews have highlighted their potential for epidermal biopotential electrodes and other wearable bioelectronic applications (Gao et al., 2023). Polymeric conductors and carbon-based composites can therefore provide sufficient electrical conductivity while remaining essentially radiolucent under standard CT imaging conditions (Cierniak, 2011; Barrett and Keat, 2004; Katsura et al., 2018; Kramme, 2011; Sawhney, 2007; Boas and Fleischmann, 2012).

Recent reviews of dry and textile ECG electrodes emphasize skin conformability, low electrode–skin impedance, mechanical durability, and stable signal acquisition during prolonged or dynamic monitoring as key design requirements (Kumar et al., 2025; Vidhya et al., 2023). Recent research has explored various polymer-based and textile electrodes for biopotential sensing (Luong et al., 2013; Stejskal and Gilbert, 2002; Bajgar et al., 2016; Patil and Deogaonkar, 2012; Bober et al., 2015; Yaghoubidoust et al., 2014; Nan et al., 2025; Lee et al., 2025). PPy and PANI coatings on cotton or carbon fabrics demonstrate good conductivity and mechanical flexibility, although durability after repeated cleaning remains a challenge (Bajgar et al., 2016; Bober et al., 2015). Composites such as PPy/GO cotton and PANI nanofiber coatings have also been proposed for smart textiles and flexible biosensors (Yaghoubidoust et al., 2014; Cetiner, 2014; Bong et al., 2019). Conducting polymers can also be polymerized on thin films for implantable applications, where radiolucency and biocompatibility are desirable (Fu et al., 2021; Shan et al., 2020; Yan et al., 2018).

Other alternatives include textile electrodes based on conductive metallic fibers (Kralikova et al., 2023; Niu et al., 2025), and MXene-based materials (Alex et al., 2024; Peng et al., 2022; Liu et al., 2023). While these exhibit low impedance and good comfort, their inclusion of transition metals (e.g., Ti) can still produce CT streaking artifacts. Recent non-contact or capacitive ECG systems (Zheng and Tang, 2025; Kafková et al., 2025; Nigusse et al., 2021; Chi et al., 2010) have improved patient comfort but require complex signal processing to suppress motion and contact artifacts.

In our recent work (Bajgar et al., 2016; Patil and Deogaonkar, 2012; Bober et al., 2015), we extended the use of PANI and PPy polymerization to fabricate radiolucent polymer-based electrode-leadwire prototype suitable for standard surface ECG sensing. The present study focuses on the complete polymer-based ECG measurement chain, covering both electrodes and leadwires. Unlike prior studies, which mainly reported laboratory electrochemical results or implantable systems (Peng et al., 2016; Mascia et al., 2023), this work provides a comprehensive evaluation of real-time ECG acquisition in a CT-simulated environment, including table motion and artifact analysis. This work goes beyond prior studies by providing a complete polymer-based ECG acquisition chain, experimentally validated under simulated CT conditions, combining.

1. Fully dry, gel-free polymer electrodes with stable ECG acquisition enabled by natural skin perspiration and demonstrated long-term signal stability without gel reapplication or additional conductive layers with reliable performance during repeated hours-long monitoring sessions.
2. A fully non-metallic ECG acquisition chain comprising PPy-coated textile electrodes and PANI-coated carbon-fiber leadwires, enabling artifact-free ECG monitoring during X-ray and CT imaging.
3. Experimental validation under simulated CT table motion using complementary quantitative signal-fidelity metrics, including waveform similarity (*r*), spectral consistency (MSC), amplitude error (R-amp), signal-to-noise ratio (SNR), baseline variability, and RMSE/MAE.
4. Direct confirmation of true radiolucency of the entire electrode–leadwire assembly by CT and X-ray imaging on phantoms and a veterinary subject.
5. Original basic research translated into a low-cost, dry, reusable, and manufacturable ECG monitoring solution, formally secured by registered patents and utility models and validated here in a clinically relevant radiological monitoring context (Electrically conducting polymers, 2013; PUV, 2011; PUV, 2013).

Together, these contributions bridge the gap between laboratory polymer electrodes and their practical application for uninterrupted ECG acquisition during the investigated X-ray and CT imaging workflows.

The remainder of this paper is organized as follows. Section 2 describes the materials, fabrication procedures, and experimental methods. Section 3 presents the results, Section 4 discusses the findings and limitations, and Section 5 concludes the paper.

## 2 Materials and methods

### 2.1 Materials for prototype fabrication

For the fabrication of radiolucent ECG electrode–leadwire prototypes, several textile and carbon-based substrates were evaluated as candidate materials. Cotton fabric and woven carbon-fiber fabric were used as candidate electrode substrates. Polyester thread, linen thread, nylon thread, nylon fishing-line monofilament, technical ribbon (86% cotton, 14% polyester), and carbon-fiber bundles were evaluated as potential leadwire substrates.

Polyaniline (PANI) and polypyrrole (PPy) were used as conductive polymer coatings. The chemicals used for polymerization included aniline hydrochloride, ammonium persulfate (APS), pyrrole, iron(III) chloride (FeCl_3_), hydrochloric acid (HCl), and deionized water.

For assembly of the final electrode–leadwire prototype, polyolefin heat-shrink tubing, textile stitching, and hook-and-loop (Velcro) fastening components were used. The final prototype configuration consisted of a PPy-coated cotton textile electrode connected to a PANI-coated carbon-fiber leadwire.

Table 1 summarizes the materials used during electrode–leadwire prototype development, including candidate substrates, polymerization reagents, and assembly components.

**TABLE 1:** Materials and relevant geometric parameters used for electrode-leadwire prototype development.

Category	Materials and relevant dimensions	Purpose
Electrode substrate candidates	Cotton fabric, thickness 0.3 mm Woven carbon-fiber fabric, thickness 0.25 mm	Electrode-composite preparation and material screening
Leadwire substrate candidates	Polyester thread, Ø 0.3 mm Linen thread, Ø 1.0 mm Nylon thread, Ø 0.15 mm Nylon fishing-line Ø 0.3 mm Carbon-fiber bundle, 24K tow Technical ribbon, 86% cotton/14% polyester, 20 mm wide, thickness 0.3 mm	Leadwire-composite preparation and material screening
Conductive polymer precursors	Aniline hydrochloride; pyrrole	PANI and PPy synthesis
Oxidizing agents	Ammonium persulfate (APS); iron(III) chloride (FeCl_3_)	Oxidative polymerization
Polymerization media	Hydrochloric acid (HCl); deionized water	Polymerization and rinsing
Assembly materials	Polyolefin heat-shrink tubing; hook-and-loop (Velcro) fastener; sewing thread	Prototype assembly

### 2.2 Methods for preparing the composites for electrodes and leadwires

#### 2.2.1 Chemical oxidative polymerization procedure

The oxidative polymerization procedures were based on our previously reported method for preparing PPy- and PANI-coated cotton fabrics (Bajgar et al., 2016), with the substrate-fixation and immersion arrangements adapted in the present study to the different electrode and leadwire geometries.

PANI coatings were prepared by oxidative polymerization of aniline hydrochloride using ammonium persulfate (APS) as the oxidizing agent. The polymerization bath consisted of 0.2 M aniline hydrochloride and 0.25 M APS, corresponding to an aniline hydrochloride-to-APS molar ratio of 1:1.25.

PPy coatings were prepared by oxidative polymerization of pyrrole using iron(III) chloride (FeCl_3_) as the oxidizing agent. The polymerization bath consisted of 0.2 M pyrrole and 0.5 M FeCl_3_, corresponding to a pyrrole-to-FeCl_3_ molar ratio of 1:2.5.

For both electrode and leadwire preparation, the fixation arrangement was adapted to the substrate geometry. Polymerization baths were prepared in a volume of 1000 mL and placed in the rectangular glass dish (350 × 250 mm, outer dimensions). Cotton fabric was stretched as a single layer on a wooden frame (300 × 100 mm, outer dimensions), whereas woven carbon-fiber fabric was immersed without frame fixation or additional weighting because its rigidity and weight were sufficient to maintain its position. Thin leadwire candidates—polyester thread, linen thread, synthetic monofilament thread, and nylon fishing-line monofilament—were wound as separated, non-overlapping turns on glass frames, whereas the technical ribbon and carbon-fiber bundle were mounted on wooden frames. At the upper and lower frame-contact regions, additional spacer strands of the same substrate material were placed beneath the wound threads to reduce direct contact with the frame and improve access of the polymerization bath to the curved sections. All substrates were kept fully submerged and separated from the vessel walls to promote uniform impregnation and coating formation.

The complete process flow is summarized in Figure 1. Before addition of the oxidizing agent, the substrates mounted on supporting frames were repeatedly immersed in and withdrawn from the corresponding monomer solution several times to promote wetting of the substrate surface. They were then kept fully immersed in the monomer solution for 30 min to ensure sufficient impregnation, including the curved frame-contact regions. During polymerization, the entire supporting frame was gently moved and tilted within the reaction bath to facilitate renewal and penetration of the reaction medium in the dense, locally overlapping, and frame-contact regions. Oxidative polymerization was subsequently initiated by adding the oxidizing agent and proceeded at room temperature for 1 h for PANI-coated substrates and for 30 min for PPy-coated cotton fabric. After polymerization, all samples were rinsed in 0.2 M HCl, washed with deionized water, and dried at room temperature. After drying, the curved frame-contact and locally overlapping regions were visually inspected, and no exposed substrate areas or visible coating discontinuities were observed. The fixation and immersion arrangements are shown in Figure 2, and the resulting conductive polymer-coated composite candidates prepared from all evaluated electrode and leadwire substrate materials are shown in Figure 3.

**FIGURE 1:**
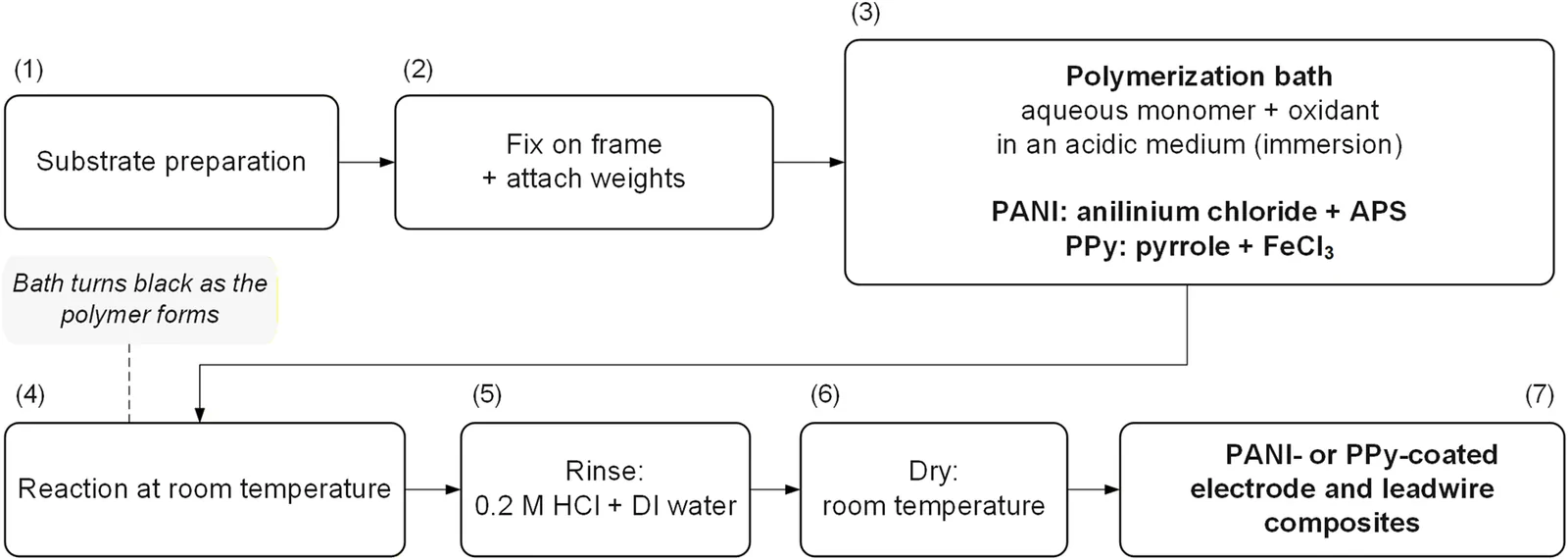
General process flow of chemical oxidative *in-situ* polymerization used for preparing conductive polymer-coated composites for electrodes and leadwires. The workflow comprises substrate preparation (1), fixation to a frame and weighting (2), immersion in an aqueous polymerization bath containing the monomer and oxidizing agent in an acidic medium (3), polymerization at room temperature (4), rinsing in 0.2 M HCl and deionized water (5), and drying at room temperature (6). The resulting composites included PANI-coated cotton fabric, PPy-coated cotton fabric, PANI-coated carbon-fiber fabric, and PANI-coated carbon-fiber bundles.

**FIGURE 2:**
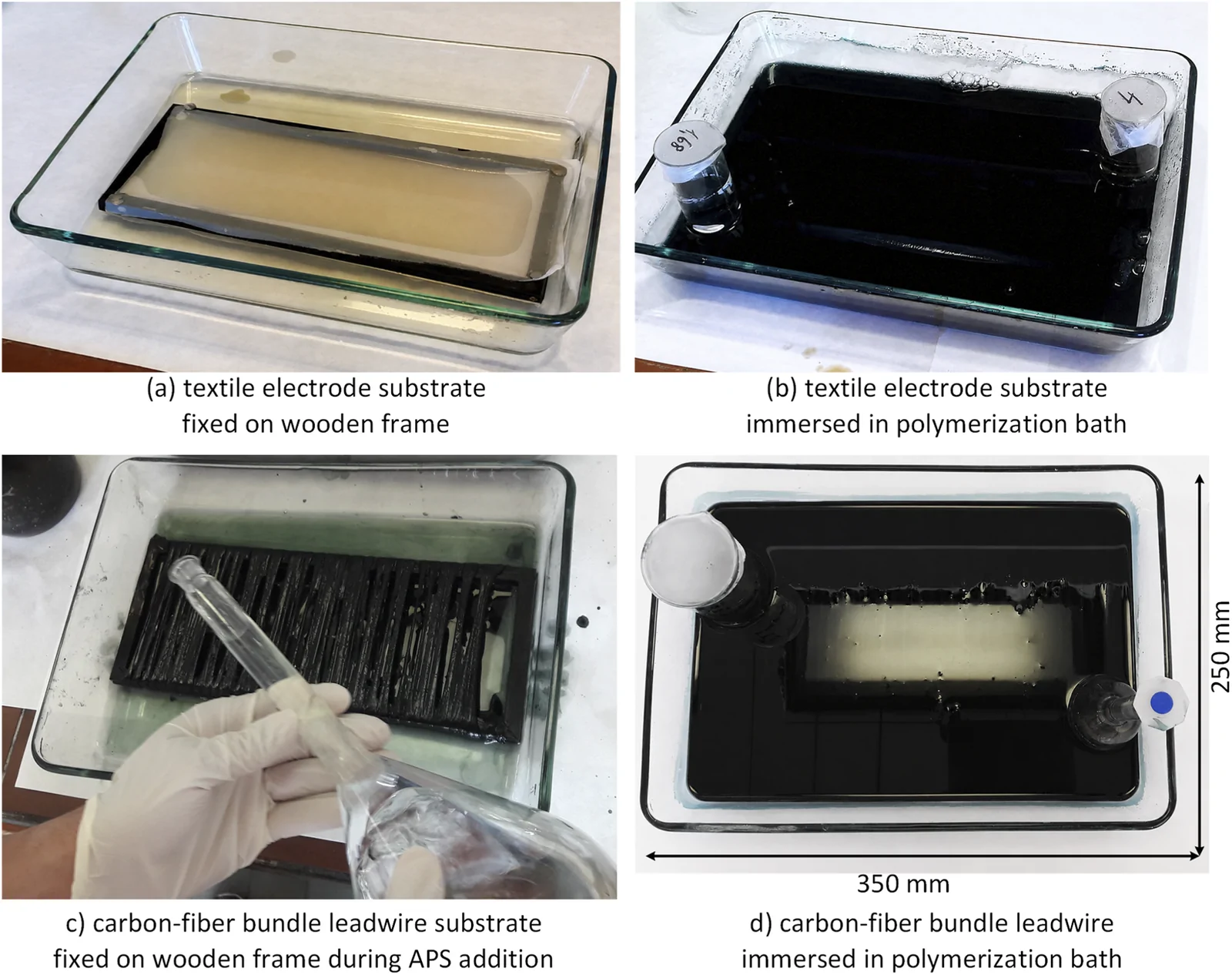
Fixation and immersion of electrode and leadwire substrates during oxidative *in-situ* polymerization **(a,b)** Cotton textile electrode substrate fixed on a wooden frame (300 mm × 100 mm, outer dimension) and immersed in the polymerization bath; the same procedure was used for PANI- and PPy-coated cotton substrates. The woven carbon-fiber fabric was immersed without frame fixation or weighting due to its sufficient rigidity and weight **(c,d)** Carbon-fiber bundle leadwire substrate fixed on a wooden frame during APS addition and immersed in the PANI polymerization bath; the same principle was used for the technical ribbon, while thin thread and monofilament substrates were mounted on a glass frame. Bath volumes shown: 1000 mL; glass dish dimensions: approximately 350 × 250 mm.

**FIGURE 3:**
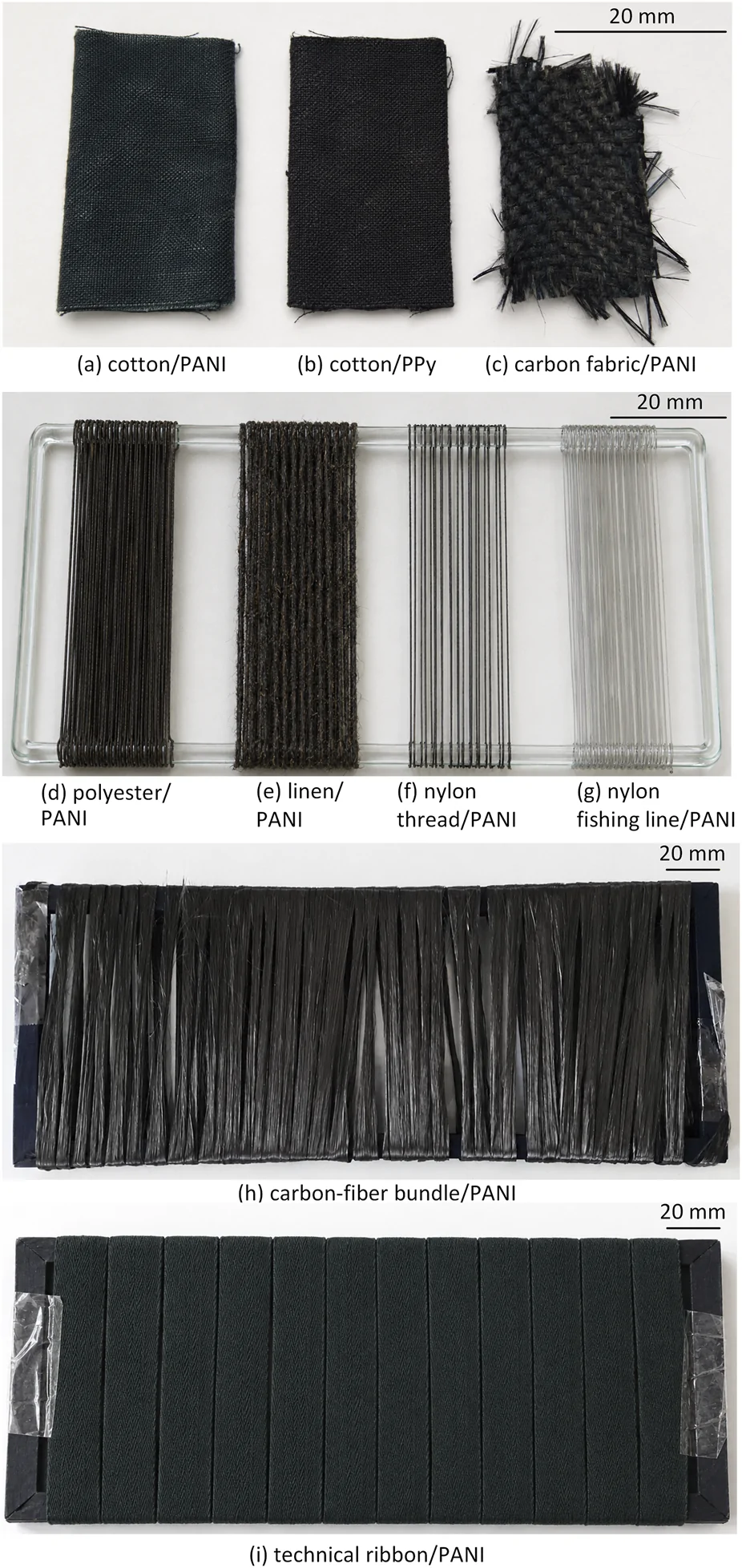
Conductive polymer-coated composite candidates prepared from all evaluated electrode and leadwire substrate materials. The electrode-related composites included **(a)** PANI-coated cotton fabric, **(b)** PPy-coated cotton fabric, and **(c)** PANI-coated carbon-fiber fabric. The leadwire-related composites included PANI-coated polyester thread **(d)**, linen thread **(e)**, nylon thread **(f)**, nylon fishing-line monofilament **(g)**, carbon-fiber bundle **(h)**, and technical ribbon **(i)**. Scale bars: 20 mm.

#### 2.2.2 Material characteristics and selection

The composites used for the final electrode–leadwire prototype were selected based on material properties, electrical characteristics, mechanical handling, ECG signal-transmission capability, and radiological compatibility. The material screening process and supporting data are summarized in Supplementary Appendix A- Material Properties of Polymer-Based Electrodes and Leadwires.

For electrode-composite evaluation, PANI-coated cotton fabric, PPy-coated cotton fabric, and PANI-coated carbon-fiber fabric were retained for quantitative comparison as standalone electrode materials (Figures 3a–c). The cotton-based PPy- and PANI-coated materials followed the same fabrication approach as reported in our previous work. In that study, representative PANI- and PPy-coated cotton samples were characterized by scanning electron microscopy (SEM), Fourier-transform infrared spectroscopy (FTIR), Raman spectroscopy, sheet-resistance measurements, and conductivity measurements of the corresponding polymer powders. These analyses confirmed successful polymer deposition, characteristic PPy/PANI spectral features, and uniform coating of the cotton fibers (Bajgar et al., 2016). Therefore, additional chemical characterization of the cotton-based coatings was not repeated in the present work. Although PANI-coated carbon-fiber fabric exhibited high electrical conductivity and was included in the standalone electrode comparison, it showed insufficient mechanical cohesion, with fraying and separation into individual fibers during handling, and was therefore not selected for the final prototype configuration.

For leadwire-composite evaluation, PANI-coated polyester thread, linen thread, synthetic monofilament thread, nylon fishing-line monofilament, technical ribbon, pure carbon-fiber bundle, and PANI-coated carbon-fiber bundle were initially considered as candidate leadwire materials (Figures 3d–i). The PANI-coated linen thread was excluded because of mechanical degradation after coating. Although the remaining thin thread and monofilament substrates retained better mechanical integrity, their high longitudinal resistance prevented reliable ECG signal transmission, and they were therefore excluded during preliminary screening. In contrast, the PANI-coated technical ribbon, pure carbon-fiber bundle, and PANI-coated carbon-fiber bundle exhibited sufficiently low longitudinal resistance and adequate mechanical integrity to advance to quantitative ECG transmission testing. The measured resistance per unit length was approximately 1.6 MΩ/m for the PANI-coated technical ribbon and approximately 11 Ω/m for the PANI-coated carbon-fiber bundle. Following the ECG transmission comparison, the 24K PANI-coated carbon-fiber bundle was selected for the final leadwire because its large effective conductive cross-section provided low longitudinal resistance, while its numerous fine filaments preserved flexibility and conformability after PANI deposition. Measurements performed on consecutive sections showed consistent resistance per unit length, supporting longitudinal uniformity of the conductive coating (Supplementary Appendix A- Material Properties of Polymer-Based Electrodes and Leadwires). The suitability of PANI-coated carbon-fiber substrates was further supported by previous reports confirming conductive-polymer deposition and characterization by SEM, FTIR, TEM, and Raman spectroscopy (Stejskal and Gilbert, 2002; Yuan et al., 2016; Sharma et al., 2009; Kim et al., 2006).

Based on the material screening, PPy-coated cotton fabric was selected as the sensing material of the final dry electrode, whereas PANI-coated carbon-fiber bundle was selected as the final leadwire component. The resulting complete electrode–leadwire prototype is described in the following subsection.

#### 2.2.3 Final electrode-leadwire prototype assembly

The final prototype was assembled using the selected material combination: a PPy-coated cotton-fabric electrode and a PANI-coated carbon-fiber leadwire. The polymerized textile was cut into 2 × 3 cm pieces, with a 2 × 2 cm area exposed as the active sensing surface.

The PANI-coated 24K carbon-fiber leadwire was mechanically protected and electrically insulated using polyolefin heat-shrink tubing, while its terminal region remained exposed for connection to the electrode. At the junction, the exposed carbon-fiber filaments were fanned out and distributed over an approximately 2 × 2 cm area on the reverse side of the polymerized textile electrode. A hook-and-loop (Velcro) backing layer was placed over the overlap region to compress the carbon-fiber bundle against the conductive textile surface and establish a broad-area electrical contact. The assembly was secured by perimeter stitching through the textile and Velcro layers, without stitching directly through or tightly constricting the conductive carbon-fiber bundle. This arrangement preserved the effective conductive cross-section, distributed mechanical stress over the contact area, maintained flexibility at the electrode–leadwire junction, and minimized potential changes in longitudinal resistance. The heat-shrink tubing provided additional strain relief and limited abrasion and excessive local bending during handling and testing. The final configuration, shown in Figure 4, was used in all subsequent experiments involving the complete electrode–leadwire prototype.

**FIGURE 4:**
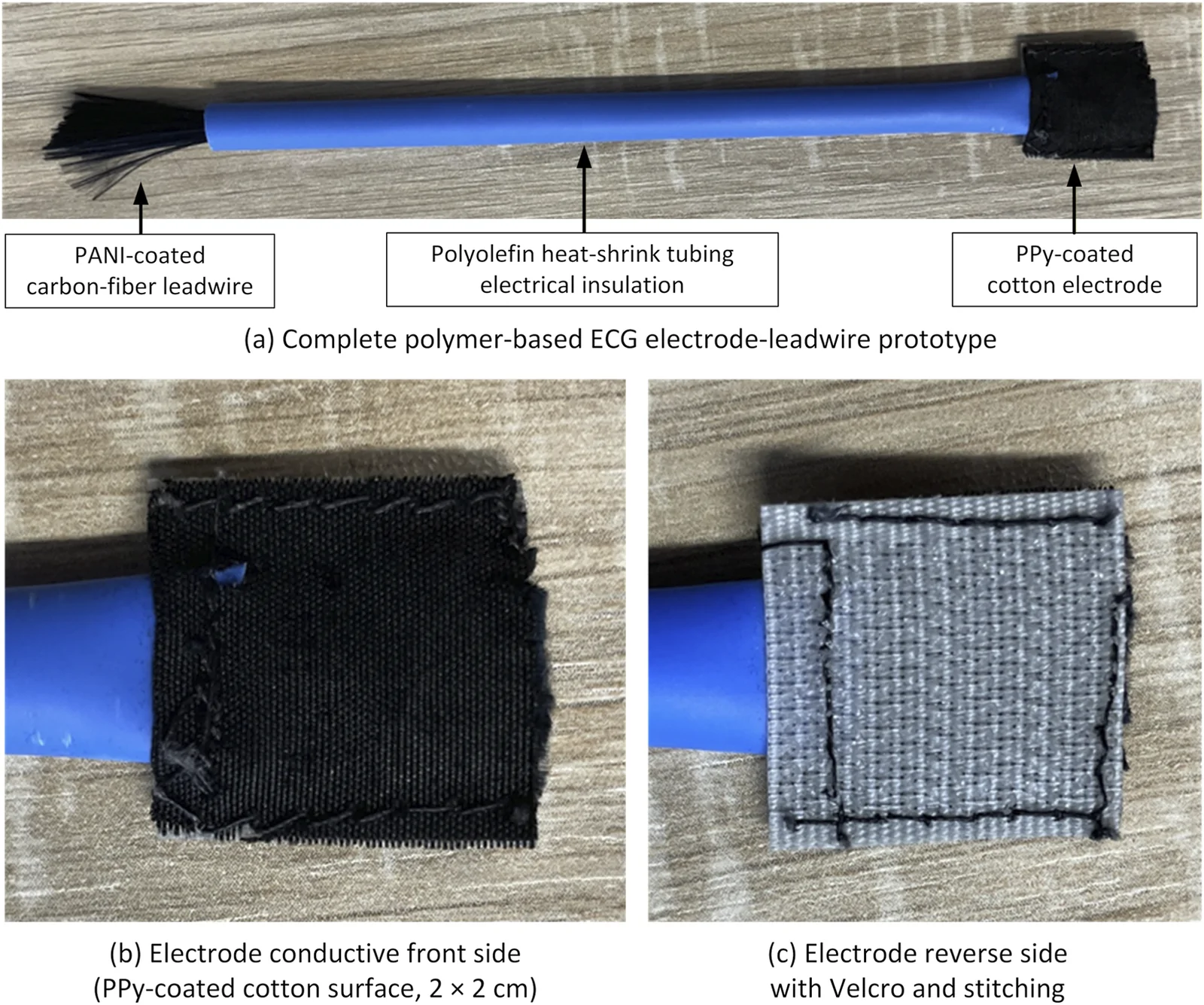
Prototype of a polymer-based PPy-coated cotton electrode integrated with a PANI-coated carbon-fiber leadwire insulated with polyolefin heat-shrink tubing. Panel **(a)** shows the complete electrode–leadwire assembly prototype with a stitched electrical junction ensuring stable electrical and mechanical contact, and a free termination for clip or connector attachment. Panel **(b)** illustrates the conductive PPy-coated cotton front side (2 × 2 cm), while panel **(c)** shows the reverse side, where the fanned-out carbon-fiber terminal is compressed against the conductive textile by the Velcro backing and secured by perimeter stitching. This assembly represents the intended core configuration of the proposed radiolucent ECG solution for CT and X-ray monitoring.

### 2.3 Data acquisition process

The experimental evaluation comprised two complementary domains: ECG signal-fidelity assessment and radiological compatibility assessment. ECG signal fidelity was evaluated through three complementary pathways: human electrode comparison, simulator-based leadwire transmission testing, and complete-system testing during simulated CT-table motion, as described in Sections 2.4.1, 2.3.2 and 2.3.3. Radiological compatibility was evaluated separately using CT phantom imaging and veterinary X-ray imaging, as described in Sections 2.3.4 and 2.4.2.

#### 2.3.1 ECG signal-fidelity evaluation

The ECG signal-fidelity assessment followed a stepwise design intended to separate the contributions of the electrode–skin interface, leadwire conductor material, and mechanical motion.

1. **Scenario 1** – assessment of polymer-based electrodes Polymer-based dry electrodes were compared with reference Ag/AgCl gel electrodes using simultaneous, time-synchronized Lead I recordings from a human subject in standard limb and Mason–Likar configurations. The complete procedure is described in Section 2.4.1.
2. **Scenario 2** – assessment of polymer-based leadwires ECG signals generated by a standardized simulator were transmitted sequentially through polymer-based and reference metallic leadwires under identical conditions, thereby isolating the influence of conductor material. The complete procedure is described in Section 2.3.2.
3. **Scenario 3** – assessment of the complete polymer-based ECG system during simulated CT-table motion. The assembled PPy-coated electrode/PANI-coated carbon-fiber leadwire system was evaluated by comparing pre-motion and in-motion segments from a single continuous three-lead recording acquired during CT-table translation. The complete procedure is described in Section 2.3.3.

These complementary scenarios separately evaluated electrode sensing quality, leadwire transmission fidelity, and complete-system stability under mechanical motion. The corresponding signal paths, acquisition modes, and scenario-specific analyses are summarized in Figure 5.

**FIGURE 5:**
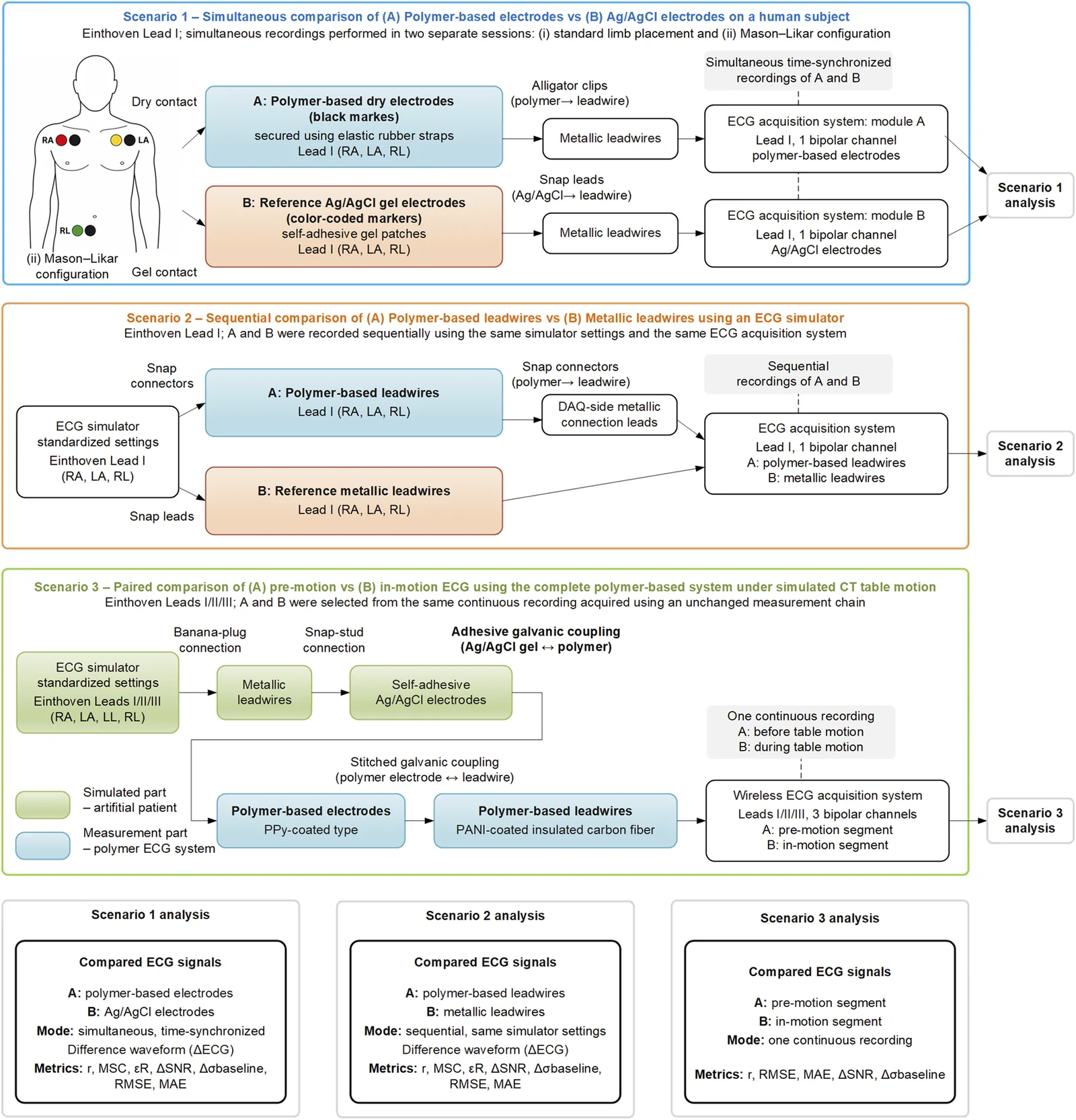
Schematic overview of the three experimental scenarios used to evaluate ECG signal fidelity of the polymer-based electrode–leadwire system and the corresponding offline analyses: (1) Scenario 1: Simultaneous, time-synchronized Lead I recordings were obtained from a human subject in two separate sessions—standard limb and Mason–Likar placements—using **(A)** polymer-based dry electrodes and **(B)** reference Ag/AgCl gel electrodes. (2) Scenario 2: Lead I signals transmitted through **(A)** polymer-based and **(B)** reference metallic leadwires were recorded sequentially under identical ECG-simulator settings using the same acquisition system. (3) Scenario 3: Paired ECG signals were selected from a single continuous three-lead recording acquired with the complete polymer-based system during simulated CT table motion **(A)** pre-motion and **(B)** in-motion. Analyses for Scenarios 1 and 2 included the difference waveform (ΔECG), Pearson correlation coefficient (r), magnitude-squared coherence (MSC), relative R-wave amplitude error (εR), signal-to-noise ratio difference (ΔSNR), baseline-variability difference (Δbaseline), root-mean-square error (RMSE), and mean absolute error (MAE). Scenario 3 was evaluated using r, RMSE, MAE, ΔSNR, and Δbaseline.

#### 2.3.2 Leadwire transmission test

Scenario 2 was designed to isolate the influence of leadwire material on ECG signal transmission. The overall workflow is summarized in Figure 5 and the experimental arrangement is shown in Figure 6.

**FIGURE 6:**
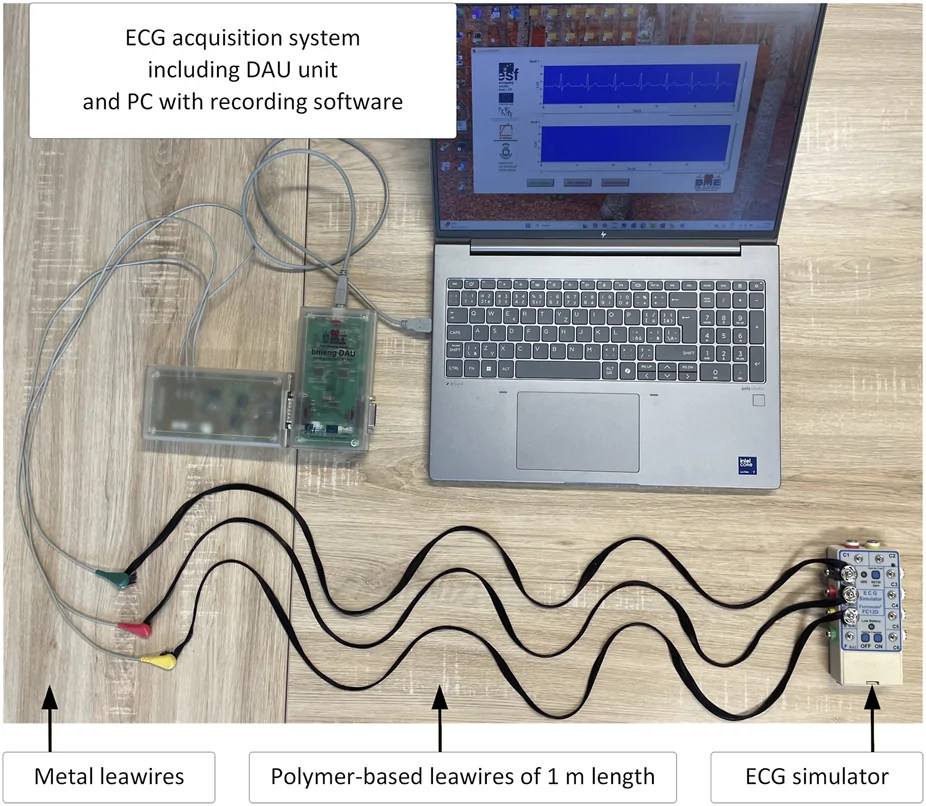
Experimental setup for Scenario 2 used to evaluate ECG signal transmission through candidate leadwire materials. A standardized Lead I ECG waveform generated by the Ferronato FC12D simulator was recorded sequentially through 1 m polymer-coated or carbon-fiber leadwire sets and through a reference metallic leadwire set using the same acquisition system and unchanged simulator settings. Each candidate recording was compared with the metallic-reference recording to assess leadwire transmission fidelity.

A standardized ECG waveform was generated using a 12-lead ECG simulator (*Ferronato FC12D*) operating at 60 beats per minute, thereby providing a stable and reproducible signal source without biological variability

A bipolar Einthoven Lead I configuration was used, comprising RA, LA, and RL conductors. All measurements were performed sequentially using the same ECG acquisition system and unchanged simulator settings.

The reference recording was first acquired using a complete set of conventional metallic leadwires. The metallic set was subsequently replaced by 1 m candidate leadwire sets comprising PANI-coated polyester thread, linen thread, nylon thread, nylon fishing-line monofilament, technical ribbon, and carbon-fiber bundles. An uncoated carbon-fiber bundle was additionally tested to determine the effect of PANI polymerization on ECG signal transmission. Each leadwire material was tested as a complete RA–LA–RL set under identical conditions.

Recordings obtained through the candidate leadwire sets were compared with the reference recording acquired through the metallic leadwires. Configurations providing stable measurable signals were retained for quantitative comparison. ECG signal fidelity was assessed using the processing procedures and quantitative metrics defined in Section 2.3.5, including waveform similarity, spectral coherence, R-wave amplitude agreement, signal-to-noise ratio, residual baseline variability, RMSE, and MAE.

#### 2.3.3 Complete-system test during CT-table motion using an artificial-patient setup

Scenario 3 evaluated whether CT-table translation affected ECG signal fidelity of the complete polymer-based electrode–leadwire prototype. The tested system comprised PPy-coated cotton electrodes and PANI-coated insulated carbon-fiber leadwires arranged according to the Mason–Likar chest configuration for acquisition of the three bipolar Einthoven leads (I–III).

To avoid exposure of a human subject to ionizing radiation, an artificial patient signal-transfer arrangement was used. An ECG simulator (*Ferronato FC12D*) operating at a constant heart rate of 60 beats per minute (BPM) was connected through conventional metallic leadwires to four self-adhesive Ag/AgCl electrodes representing section of the artificial patient (RA, LA, LL, and RL). Each Ag/AgCl electrode was galvanically coupled face-to-face with the corresponding PPy-coated cotton electrode through a conductive gel interface and mechanically secured using medical adhesive tape. The polymer electrodes were connected through PANI-coated insulated carbon-fiber leadwires to a wireless ECG recorder (*Corscience ECG BT12*), thereby forming the complete measurement section of the proposed system. The experimental arrangement is shown in Figure 7.

**FIGURE 7:**
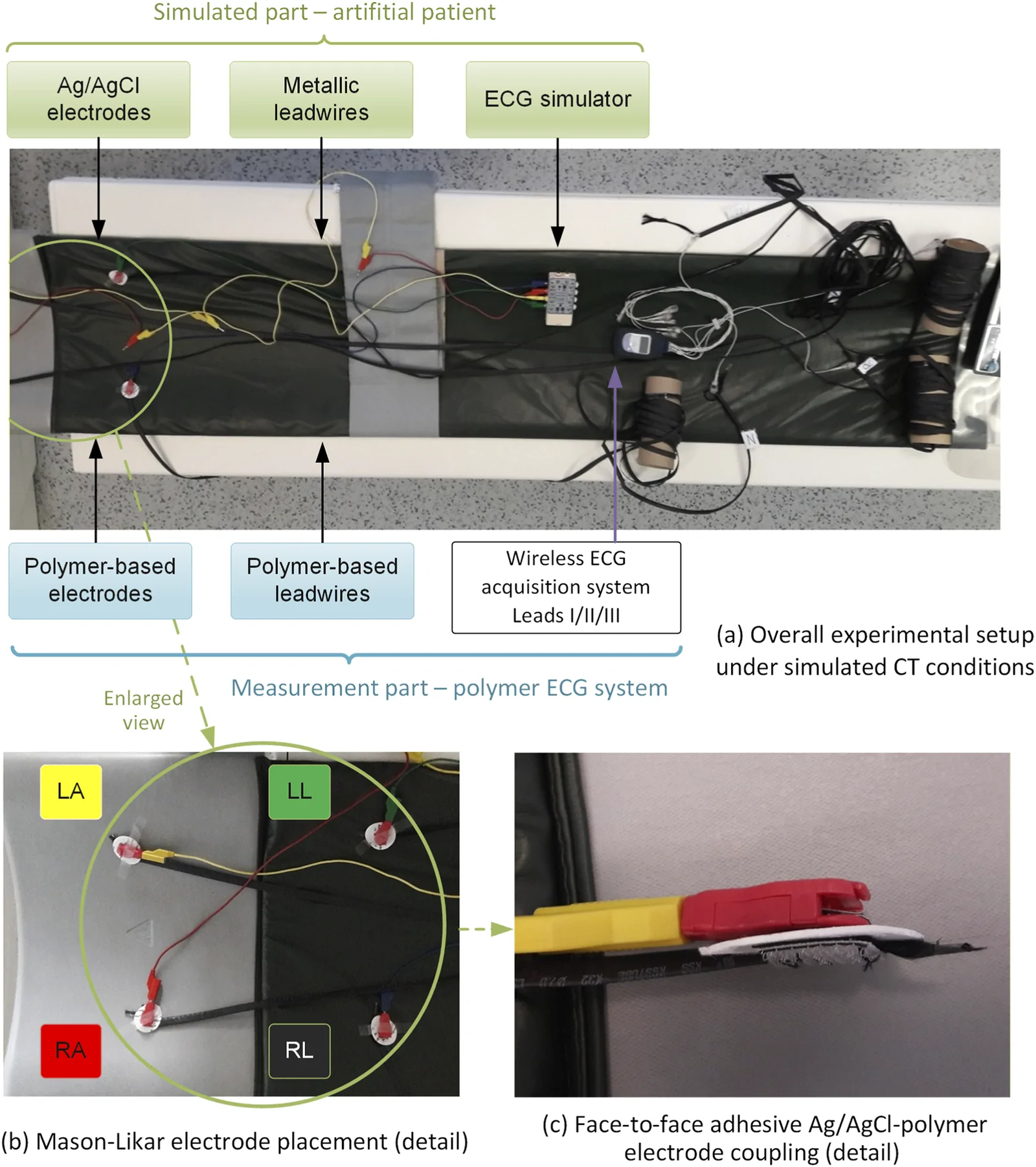
Experimental setup for Scenario 3 used to evaluate ECG signal fidelity during simulated CT-table motion. **(a)** Overall measurement chain consisting of the artificial-patient section—ECG simulator, metallic leadwires, and self-adhesive Ag/AgCl electrodes—and the polymer-based measurement section comprising PPy-coated cotton electrodes, PANI-coated insulated carbon-fiber leadwires, and the wireless ECG recorder. **(b)** Mason–Likar placement of the RA, LA, LL, and RL electrode pairs on the CT table. **(c)** Detail of the face-to-face galvanic coupling between the Ag/AgCl and PPy-coated electrodes through the conductive gel interface.

The experiment was performed using a CT scanner (*Siemens SOMATOM Definition AS*) with an abdominal protocol selected in consultation with radiology staff (120 kV, 100 mAs, CTDIvol = 0.81 mGy, DLP = 36 mGy·cm). The protocol was used to reproduce clinically representative CT-table translation.

ECG signals were recorded continuously without changing the simulator output or measurement chain. For each bipolar Einthoven lead, a segment acquired immediately before table motion (A) and a corresponding segment acquired during table motion (B) were selected from the same continuous recording. Motion-related signal degradation was evaluated using the processing procedures and quantitative metrics defined in Section 2.3.5, including Pearson correlation coefficient, RMSE, MAE, ΔSNR, and Δσbaseline.

#### 2.3.4 CT phantom imaging and radiolucency assessment

The radiolucency of the proposed polymer-based ECG electrode–leadwire prototype assembly was evaluated under controlled CT and X-ray imaging conditions and compared directly with a conventional Ag/AgCl electrode–metallic-leadwire system. The polymer-based configuration comprised PPy-coated cotton electrodes connected to PANI-coated carbon-fiber leadwires, representing the complete non-metallic patient-side ECG assembly intended for use during radiological monitoring.

To assess the contribution of auxiliary components to CT visibility, two polymer-based leadwire variants were tested: one insulated with polyolefin heat-shrink tubing and one without insulation. The PPy-coated electrodes included the Velcro backing and stitching used in the final prototype. Conventional Ag/AgCl electrodes connected to metallic leadwires were used as the reference configuration.

Measurements were performed using two tissue-equivalent models: a HEAD-16 water-equivalent phantom with a diameter of 16 cm and a custom agar tissue-mimicking phantom measuring 16 × 3.5 × 23 cm. The HEAD-16 phantom provided a standardized water-equivalent background, whereas the agar phantom provided a homogeneous soft-tissue-like imaging background. Agarose-based gels have previously been used as brain-tissue surrogates in multimodality CT/MRI phantoms (Gallas et al., 2015).

On each phantom, the complete polymer-based electrode–leadwire configurations and the conventional Ag/AgCl electrode–leadwire configuration were positioned within the same imaging field. The electrodes were secured to the phantom surfaces using medical adhesive tape, with an inter-electrode spacing of approximately 7 cm. For each phantom, both a CT Surview acquisition and a native helical scan were obtained with all investigated configurations present under identical acquisition conditions.

This arrangement enabled direct comparison of the radiological conspicuity and artifact generation of the polymer-based and conventional metallic ECG systems. The experimental configurations are shown in Figure 8, while the corresponding CT results are presented in Section 3.2.1 (CT phantom radiolucency). CT acquisition parameters and quantitative image-evaluation methods are described in Section 2.3.5.

**FIGURE 8:**
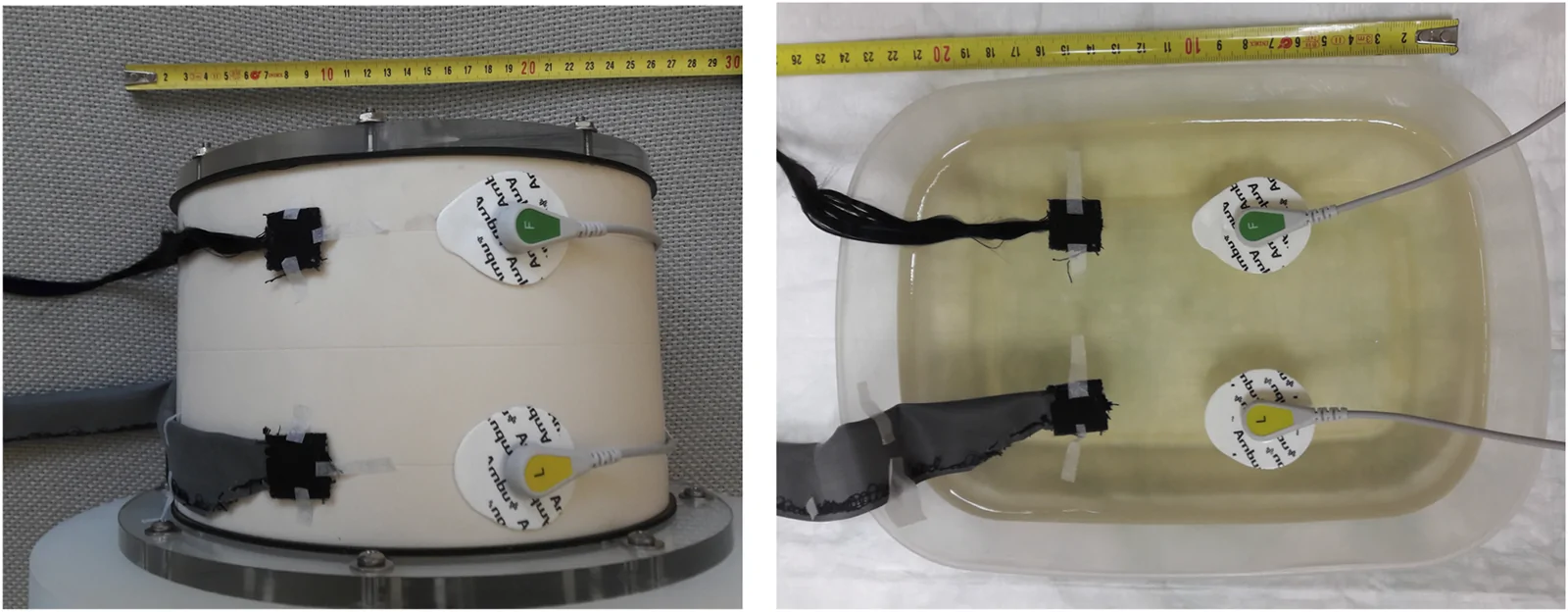
Electrode and leadwire placement used for CT radiolucency assessment. Left: HEAD-16 water-equivalent phantom. Right: agar tissue-mimicking phantom. On both phantoms, PPy-coated cotton electrodes connected to PANI-coated carbon-fiber leadwires with and without polyolefin heat-shrink insulation were compared with conventional Ag/AgCl electrodes connected to metallic leadwires. The configurations were positioned within the same imaging field to enable direct comparison of component visibility and CT artifact generation. The corresponding CT results are presented in Section 3.2.1 (CT phantom radiolucency).

#### 2.3.5 Acquisition systems and evaluation metrics

##### 2.3.5.1 ECG acquisition systems

Scenarios 1 and 2 were performed using an external optically insulated A/D converter (*VSB-BMENG DAU*) with differential inputs (RA and LA) and a passive RL reference. The input stage employed an INA126 instrumentation amplifier configured with a gain of 1500, together with a 0.5 Hz high-pass RC filter to suppress baseline drift and a simple anti-aliasing network. Signals were sampled at 2000 Hz with a 12-bit MCP3208, providing an effective input resolution of ≈0.8 µV/bit. The analog front end provided a bandwidth of 0.5–500 Hz and an input impedance of ≈1 GΩ.

In Scenarios 1, two identical, electrically independent, and time-synchronized analog front-end modules were used to acquire the polymer-based and Ag/AgCl signals simultaneously. Each module employed its own RA, LA, and RL electrodes. In Scenario 2, a single module was used for sequential acquisition through the candidate and reference metallic leadwire sets under unchanged simulator and acquisition settings.

Scenario 3 employed a wireless ECG recorder (*Corscience ECG BT12*), configured for data acquisition during CT-table motion. The device sampled ECG signals at 500 Hz, with a digital resolution of ≈2.6 µV/bit, a bandwidth of 0.05–150 Hz, and an input impedance >20 MΩ.

Both systems provided diagnostic-grade ECG acquisition, complying with international recommendations for ≤5 µV/bit resolution. System B (*Corscience ECG BT12*) implements the diagnostic 0.05–150 Hz (−3 dB) bandwidth, whereas System A (*VSB-BMENG DAU*) uses a 0.5 Hz high-pass to suppress baseline wander, consistent with monitoring/ambulatory practice (IEC 60601-2-27/-47).

##### 2.3.5.2 ECG signal-processing and fidelity metrics

To compare polymer-based electrodes and leadwires with the Ag/AgCl reference and conventional metal leadwires, we combined a visual “difference-trace” check with quantitative time- and frequency-domain metrics computed on the raw acquired signals (i.e., as digitized by the front ends described above, including the common 0.5 Hz high-pass and anti-alias path).

For the comparative visualization (ΔECG), we formed a difference waveform for every 10 s segment as defined in Equation 1:

$$\Delta \text{ECG}\left(t\right)=x_{\text{poly}}\left(t\right)-x_{\text{ref}}\left(t\right),$$ (1)

after removing each signal’s constant offset (mean value) to avoid trivial DC shifts.

Amplitude agreement was evaluated using R-peak amplitudes detected in both signals within the same beats, and the relative amplitude error was calculated according to Equation 2:

$$\varepsilon_{R}\;\left(\%\right)=100\cdot \frac{R_{\text{poly}}-R_{\text{ref}}}{R_{\text{ref}}}.$$ (2)

The time-domain morphology for waveform similarity assessment was quantified by Pearson’s linear correlation coefficient *r*, calculated according to Equation 3 (Orphanidou and Orphanidou, 2018):

$$r=\frac{\sum_{i=1}^{N}\left(x_{i}-\bar{x}\;\right)\left(y_{i}-\bar{y}\;\right)}{\sqrt{\sum_{i=1}^{N}{\left(x_{i}-\bar{x}\;\right)}^{2}}\sqrt{\sum_{i=1}^{N}{\left(y_{i}-\bar{y}\;\right)}^{2}}},$$ (3)

where *N* is samples count, *x*_*i*_ and *y*_*i*_ denote individual samples of the polymer and reference signals, respectively. The coefficient *r* ranges from −1 to +1, expressing the degree of linear similarity between two waveforms, where values close to +1 indicate strong similarity in shape and timing.

For frequency-domain consistency, spectral similarity was assessed using the magnitude-squared coherence (MSC), defined in Equation 4 from the cross- and auto-power spectral densities (PSDs) as (Welch, 1967):

$$C_{xy}\left(f\right)=\frac{{\left|P_{xy}\left(f\right)\right|}^{2}}{P_{xx}\left(f\right)P_{yy}\left(f\right)}\in \left[0,1\right],$$ (4)

where *P*_*xy*_(*f*) is the cross-power spectral density and *P*_*xx*_(*f*), *P*_*yy*_(*f*) are the autospectra of the signals. Coherence values close to 1 indicate strong linear coupling at frequency *f*, while values near 0 indicate no consistent relationship. PSDs were estimated with Welch’s method (Hamming window, 3 s, 50% overlap). We summarize frequency-domain agreement by the band-mean MSC over 0.5–40 Hz, which covers the dominant energy of the QRS complex and most clinically relevant ECG content (Tereshchenko and Josephson, 2015; Kligfield et al., 2007).

Together, ΔECG plots reveal local differences, Pearson’s *r* captures overall morphology similarity, R-amp error evaluates amplitude agreement of the R-peak and band-mean MSC quantifies frequency-domain consistency across the clinically relevant 0.5–40 Hz band.

For the evaluation of motion artifacts induced by CT table translation, we focused on the ECG band of 1–25 Hz, which covers the diagnostically relevant P–QRS–T components while also including the frequency range where motion-induced disturbances are most pronounced. In this context, frequency-domain coherence was not applied, as table motion produces transient distortions rather than stable spectral differences. Instead, waveform distortion was quantified in the time domain using Pearson’s correlation coefficient to assess overall morphology similarity, complemented by mean absolute error (MAE) and root mean square error (RMSE), calculated according to Equation 5, to capture local amplitude deviations between pre- and in-motion segments:

$$\text{MAE}=\frac{1}{N}\sum_{i=1}^{N}\left|x_{i}-y_{i}\right|,\text{RMSE}=\sqrt{\frac{1}{N}\sum_{i=1}^{N}{\left(x_{i}-y_{i}\right)}^{2}}$$ (5)

For offline signal processing, finite impulse response (FIR) filters were applied with passbands tailored to the evaluation tasks: 0.5–40 Hz prior to coherence analysis and 1–25 Hz for motion-artifact assessment during CT table translation. FIR filters were chosen for their linear phase response, preserving the phase morphology of the P–QRS–T complexes. The design provided ≥60 dB stopband attenuation and passband ripple <0.01 dB, thereby minimizing distortion within the clinically relevant frequency range.

Signal-to-noise ratio (SNR) was computed separately for each 10 s ECG recording on a beat-to-beat basis according to Equation 6:

$$\text{SNR}=\frac{1}{N}\sum_{i=1}^{N}20\;\log_{10}\left(\frac{A_{i}^{\text{QRS}}}{\sigma_{i}^{\text{noise}}}\right),\Delta \text{SNR}={\text{SNR}}_{\text{poly}}-{\text{SNR}}_{\text{ref}},$$ (6)

where *i* denotes the heartbeat index, *N* is the number of detected heartbeats in the recording, A_*i*_^QRS^ is the peak-to-peak amplitude of the QRS complex, and *σ*_*i*_^noise^ is the standard deviation of the signal in the corresponding 80 m pre-QRS isoelectric window. ΔSNR expresses the difference in signal-to-noise ratio between the polymer-based electrode/leadwire and Ag/AgCl/metallic reference, or the in-motion and pre-motion segment in case of simulated CT table motion.

Baseline stability was evaluated separately for each 10 s ECG recording using beat-to-beat estimates of the isoelectric baseline level. For each detected heartbeat, the baseline value was obtained as the median signal level within the corresponding pre-QRS isoelectric window of 80 m duration. Baseline stability was quantified as the standard deviation of these baseline estimates according to Equation 7:

$$\sigma_{\text{baseline}}=\sqrt{\frac{1}{N-1}\sum_{i=1}^{N}{\left(b_{i}-\bar{b}\right)}^{2}},\Delta \sigma_{\text{baseline}}=\sigma_{\text{baseline},\text{poly}}-\sigma_{\text{baseline},\text{ref}}$$ (7)

here, *b*_*i*_ denotes the baseline level of the *i*th heartbeat within the given 10 s ECG recording, *b̄* is the mean baseline level across all detected heartbeats, and *N* is the number of detected heartbeats. Δσ_baseline_ expresses the difference in baseline stability between the polymer-based electrode/leadwire and Ag/AgCl/metallic reference, or the in-motion and pre-motion segment in case of simulated CT table motion. Due to the presence of a 0.5 Hz hardware high-pass filter in the acquisition chain, this metric characterizes residual beat-to-beat baseline variability rather than low-frequency baseline wander below the filter cutoff.

##### 2.3.5.3 CT image acquisition system

Phantom imaging was performed using a CT scanner (*Philips Brilliance 64*). On each phantom, the polymer-based and conventional metallic configurations were positioned simultaneously within the same imaging field and were therefore acquired under identical scanner conditions. Both a CT Surview acquisition and a native helical scan were obtained without contrast administration.

Scanner acquisition parameters, display window settings, and scanner-reported dose indices are summarized in Table 2. The native helical images were evaluated using a standard soft-tissue display setting of W/C = 320/40 and an additional high-contrast setting of W/C = 1600/−500 to enhance the visual conspicuity of low-density auxiliary components, including the Velcro backing and polyolefin leadwire insulation. Images were displayed and evaluated using AMIS PACS Media Lite v3.2 software (ICZ a.s.), including W/C adjustment, image magnification, distance measurement, ROI placement, and annotation.

**TABLE 2:** CT acquisition parameters, display window settings, and scanner-reported dose indices for HEAD-16 and agar phantom imaging.

Phantom type	Scan type	W/C^1^	Q^2^ (mA·s)	U^3^ (kV)	CTDIvol^4^ (mGy)	DLP^5^ (mGy·cm)
HEAD 16	Surview	1500/20	1	120	0.15	5.2
HEAD 16	NATIV helical	320/40 1600/− 500	300	120	38.30	990.1
Agar	Surview	1600/− 500	1	120	0.15	5.2
Agar	NATIV helical	320/40 1600/− 500	299	120	38.30	1191.8

1) W/C (Window Width and Window Center). 2) Q (Applied tube charge). 3) U (Applied tube voltage). 4) CTDIvol (Computed Tomography Dose Index–volumetric) (mGy). 5) DLP (Dose Length Product) (mGy·cm).

##### 2.3.5.4 CT image evaluation metrics

The radiological conspicuity of the polymer-based electrodes, leadwires, and auxiliary materials was evaluated quantitatively on reconstructed CT images using Hounsfield-unit measurements (Emonde et al., 2024). Regions of interest (ROIs) were manually positioned within the investigated component and in the adjacent phantom background while avoiding material edges and partial-volume regions. HU measurements were obtained from the reconstructed image data and were independent of the display window settings used for visual inspection.

The mean CT number, expressed in Hounsfield units (HU), within each ROI was calculated using standard ROI-based CT-number analysis (Samei et al., 2019). The relative attenuation difference, ΔHU was subsequently defined in the present study as the difference between the mean HU value of the investigated material and that of the surrounding reference medium, as given in Equation 8:

$${\bar{\text{HU}}}_{\text{mat}}=\frac{1}{N}\sum_{i=1}^{N}{\text{HU}}_{i},\Delta \text{HU}={\bar{\text{HU}}}_{\text{mat}}-{\bar{\text{HU}}}_{\text{ref}}$$ (8)

where ${\text{HU}}_{i}$ represents the CT number of the *i*th voxel within the ROI and *N* is the total number of voxels, and ${\bar{\text{HU}}}_{\text{ref}}$ is the mean CT number of the adjacent phantom background ROI. The magnitude of the attenuation difference, |ΔHU|, was used to characterize contrast between the investigated component and the surrounding phantom. Smaller |ΔHU| values indicate lower local contrast and therefore lower radiological conspicuity relative to the background. Components exhibiting |ΔHU| values close to zero are therefore expected to be radiologically inconspicuous relative to the local phantom background.

For descriptive classification in the present study, components that were not visually distinguishable under the evaluated window settings and exhibited |ΔHU| < 40 HU were classified as invisible. Components with |ΔHU| values of 40–100 HU that became only faintly discernible under high-contrast windowing were classified as slightly visible, whereas components with |ΔHU| > 100 HU were classified as visible. These operational categories were used to support the visual comparison and should not be interpreted as universal clinical thresholds for CT artifact generation.

### 2.4 *In vivo* validation

*In vivo* validation comprised two complementary experiments: human-subject assessment of ECG signal fidelity using standalone polymer-based electrodes and veterinary X-ray assessment of the radiographic visibility of the complete electrode–leadwire prototype.

#### 2.4.1 Human ECG validation of polymer-based electrodes

In Scenario 1, polymer-based electrodes were evaluated against standard Ag/AgCl gel electrodes in a single adult volunteer using bipolar Einthoven Lead I. Measurements were performed in two separate sessions using standard limb placement and the Mason–Likar chest configuration. ECG signals were acquired simultaneously using two independent, time-synchronized acquisition modules: one connected to the polymer-based electrodes and the other to the Ag/AgCl reference electrodes. Each system employed its own differential input pair (RA and LA) and an independent passive reference electrode (RL).

The evaluated polymer electrodes comprised PANI-coated cotton fabric, PPy-coated cotton fabric, and PANI-coated carbon-fiber fabric, prepared as described in Section 2.2. Each electrode comprised a 2 × 2 cm sensing area and a separate 2 × 1 cm connection tab.

For the Mason–Likar recordings, the polymer electrodes were positioned at the corresponding Lead I locations and secured using Velcro-fastened Esmarch rubber straps to provide stable contact pressure and reduce motion artifacts. The polymer electrodes were connected to conventional metallic leadwires using insulated alligator clips secured to the skin with adhesive tape, while self-adhesive Ag/AgCl electrodes were positioned in close proximity to enable simultaneous comparison.

For standard limb-lead recordings, paired polymer and Ag/AgCl electrodes were positioned in close proximity on the distal right and left upper limbs and the right lower limb. The polymer electrodes were secured using electrically insulated clamps to provide stable and comparable contact pressure. Both electrode configurations were connected to their respective acquisition modules using the same type of conventional metallic leadwires. The experimental arrangements are shown in Figure 9.

**FIGURE 9:**
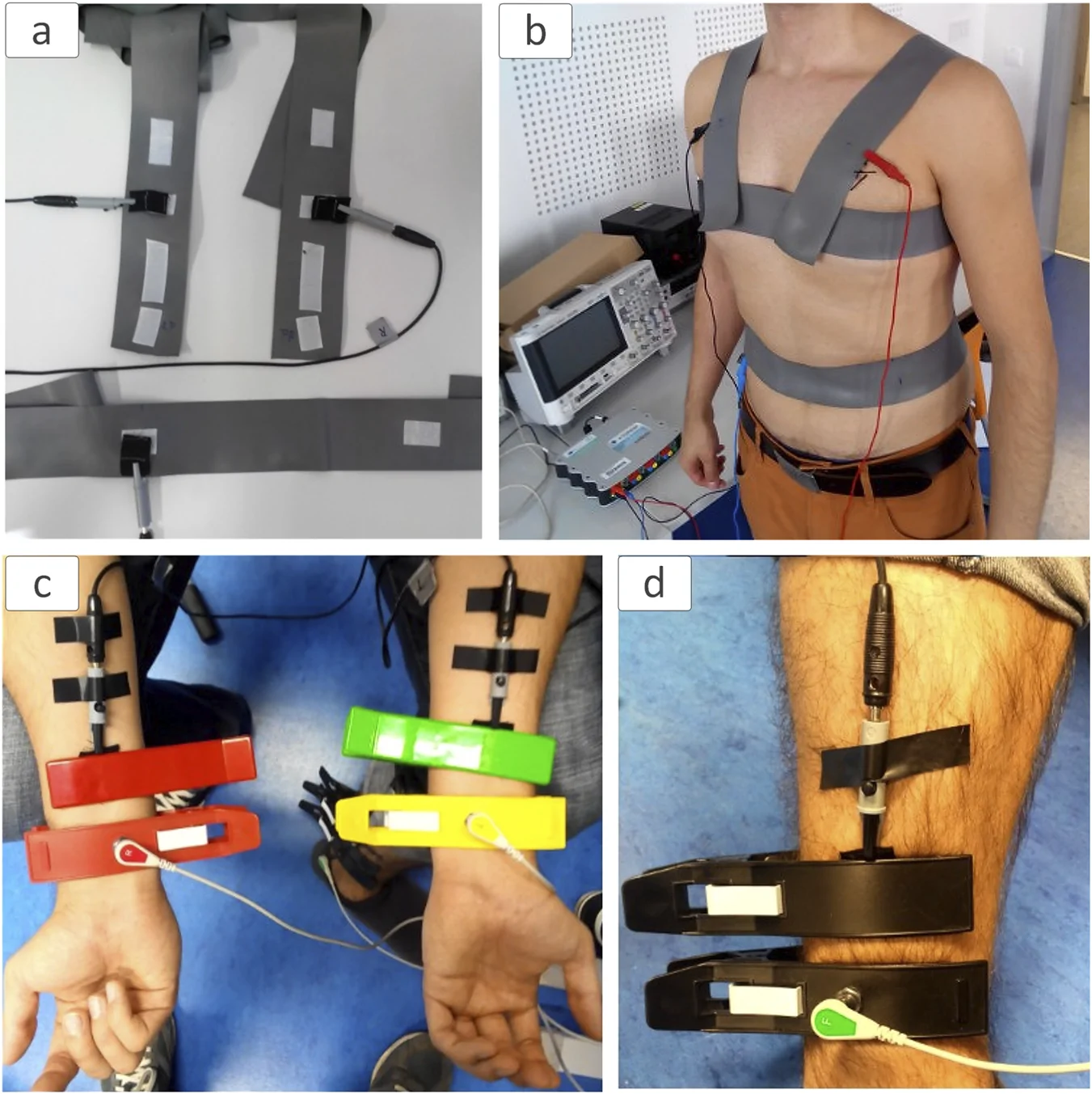
Human-subject setup used for Scenario 1. **(a)** Esmarch rubber straps equipped with polymer-based electrodes and insulated clip connections. **(b)** Polymer-based placement in the Mason–Likar configuration **(c,d)** Paired polymer-based and Ag/AgCl electrode placement on the distal limbs for standard Lead I acquisition. The two electrode configurations were connected to independent, time-synchronized ECG acquisition modules.

ECG signal fidelity was evaluated using the signal-processing procedures and quantitative metrics defined in Section 2.3.5.

#### 2.4.2 Veterinary X-ray validation of the complete prototype

The radiographic visibility of the complete polymer-based prototype was evaluated *in vivo* using an anaesthetized veterinary subject (cat). The tested configuration comprised PPy-coated cotton electrodes connected to PANI-coated carbon-fiber leadwires insulated with polyolefin heat-shrink tubing. Conventional Ag/AgCl electrodes connected to metallic leadwires were used as the reference configuration.

Both configurations were positioned over the thoracic region within the same planned imaging field and secured using medical adhesive tape to prevent displacement during image acquisition. This arrangement enabled direct comparison of the radiographic conspicuity and artifact generation of the polymer-based and conventional metallic ECG components under identical X-ray conditions. The electrode placement is shown in Figure 10, and the resulting radiographic findings are presented in Section 3.2.2 (Veterinary X-ray radiolucency).

**FIGURE 10:**
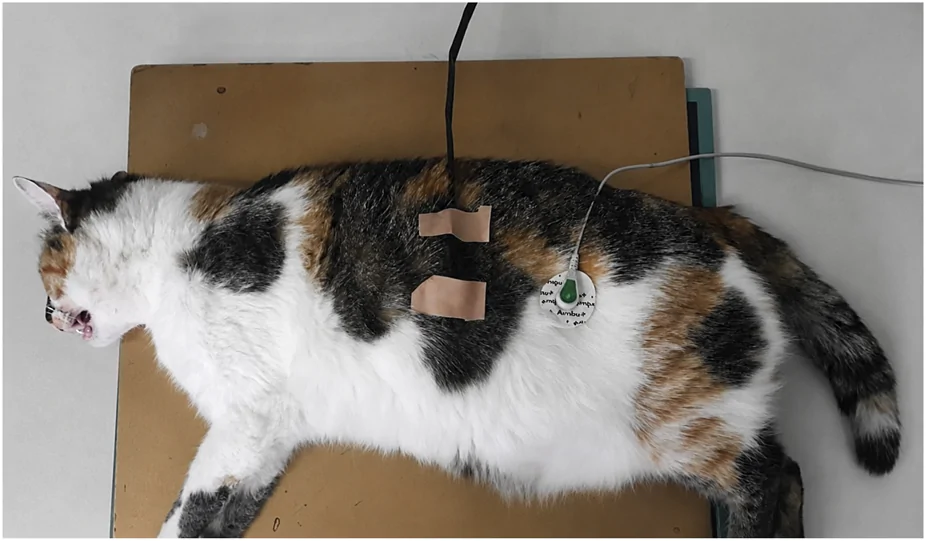
Placement of the electrode–leadwire configurations on the veterinary subject before X-ray imaging. The polymer-based configuration comprised PPy-coated cotton electrodes connected to PANI-coated carbon-fiber leadwires insulated with polyolefin heat-shrink tubing. Conventional Ag/AgCl electrodes connected to metallic leadwires served as the reference. Both systems were positioned over the thoracic region within the imaging field used for the radiographic assessment described in Section 3.2.2 (Veterinary X-ray radiolucency).

X-ray imaging was performed using a mobile radiographic system (*Siemens POLYMOBIL 10*) operated at 81 kV and 5 mA·s. The veterinary experiment was used solely to assess radiographic visibility and artifact generation; ECG signal fidelity was not evaluated in the animal subject.

## 3 Results

The results are organized according to the two complementary evaluation domains defined in Section 2.3: ECG signal fidelity and radiological compatibility. ECG performance is presented sequentially for standalone electrodes, standalone leadwires, and the complete electrode–leadwire prototype during CT-table motion. Radiological compatibility is subsequently evaluated using CT phantom and veterinary X-ray imaging. Within each subsection, quantitative performance results are presented first, followed by the corresponding representative signal or imaging data.

### 3.1 ECG signal-fidelity results

#### 3.1.1 Scenario 1: human ECG validation of standalone polymer-based electrodes

Standalone polymer-based dry ECG electrodes were evaluated against reference Ag/AgCl gel electrodes using simultaneous bipolar Einthoven Lead I recordings from a human subject in the standard limb and Mason–Likar configurations. The investigated materials comprised PPy-coated cotton fabric, PANI-coated cotton fabric, and PANI-coated carbon-fiber fabric. The experimental procedure and signal-processing methods are described in Sections 2.4.1 and 2.3.5, respectively. Quantitative results are summarized in Table 3.

**TABLE 3:** Quantitative comparison of polymer-based electrodes with the Ag/AgCl reference during Mason–Likar and standard limb Lead I ECG recordings.

​	Mason-likar lead I ECG				
Polymer electrode type	Pearson *r* ^1^ (−)	MSC_0.5-40_^2^ (−)	*ε*_R_^3^ (%)	ΔSNR^4^ (dB)	Δ*σ*_baseline_^5^ (µV)
PANI-coated cotton fabric	0.9332	0.8142	+10.8	−1.8	+45
PPy-coated cotton fabric	0.9515	0.8969	+12.7	+5.3	+16
PANI-coated carbon fiber fabric	0.9349	0.8775	+7.2	+0.2	+46

​	Standard limb lead I ECG				
Polymer electrode type	Pearson *r* ^1^ (−)	MSC_0.5-40_^2^ (−)	ε_R_^3^ (%)	ΔSNR (dB)	Δσ_baseline_ (µV)
PANI-coated cotton fabric	0.9944	0.9803	−1.9	−0.3	−10
PPy-coated cotton fabric	0.9935	0.9942	−3.1	−2.2	−12
PANI-coated carbon fiber fabric	0.9835	0.9843	−2.5	+0.0	+3.3

1) *r*–Pearson correlation coefficient. 2) MSC_0.5-40_ – Magnitude-squared Coherence computed in frequency band from 0.5 Hz to 40 Hz. 3) ε_R_–Relative amplitude deviation between polymer and Ag/AgCl signals, with DC components suppressed by the ECG system’s 0.5 Hz hardware high-pass filter. 4) ΔSNR–Difference in signal-to-noise ratio between the polymer-based electrode and the Ag/AgCl reference. 5) Δ*σ*_baseline_–Difference in residual baseline variability between the polymer-based electrode and the Ag/AgCl reference, quantified as the standard deviation of the pre-QRS isoelectric baseline (80 m window) after 0.5 Hz hardware high-pass filtering.

Positive values of *ε*_R_, ΔSNR, and Δ*σ*_baseline_ indicate higher amplitude, higher SNR, or increased baseline variability of the polymer-based electrode relative to the Ag/AgCl reference, respectively, while negative values indicate lower values compared to the reference. Detailed metric definitions are provided in Section 2.3.5.

Across all evaluated material–placement combinations, Pearson correlation coefficients ranged from 0.9332 to 0.9944, while band-mean MSC values ranged from 0.8142 to 0.9942. Among the tested materials, the PPy-coated cotton fabric exhibited the best overall performance, particularly in limb-lead recordings (*r* = 0.9935, MSC_0.5-40_ = 0.9942, *ε*_R_ = −3.1%), followed by PANI-coated cotton fabric and PANI-coated carbon fiber fabric. The Mason–Likar recordings generally exhibited lower correlation and coherence and larger residual baseline differences than the standard limb recordings.

Absolute SNR values ranged from approximately 36–38 dB for both the Ag/AgCl reference and polymer-based electrodes in the standard limb configuration. In the Mason–Likar configuration, Ag/AgCl SNR values ranged from approximately 38–41 dB, whereas polymer-based electrode values ranged from approximately 35–41 dB.

Residual baseline variability ranged from 15 to 25 µV for the Ag/AgCl reference and from 5 to 30 µV for the polymer-based electrodes in the standard limb configuration. In the Mason–Likar configuration, the corresponding ranges were 7–9 μV and 8–50 μV, respectively.

Figure 11 presents a representative simultaneous recording obtained using the PPy-coated cotton electrode selected for the final prototype and the Ag/AgCl reference in the standard limb configuration. The signals exhibited closely matching waveform morphology, with residual differences remaining approximately within ±0.1 mV relative to an ECG amplitude of approximately 1.5 mV. No amplitude scaling was applied. For visualization, the Ag/AgCl and PPy traces were vertically shifted by 3.0 mV and 1.5 mV, respectively.

**FIGURE 11:**
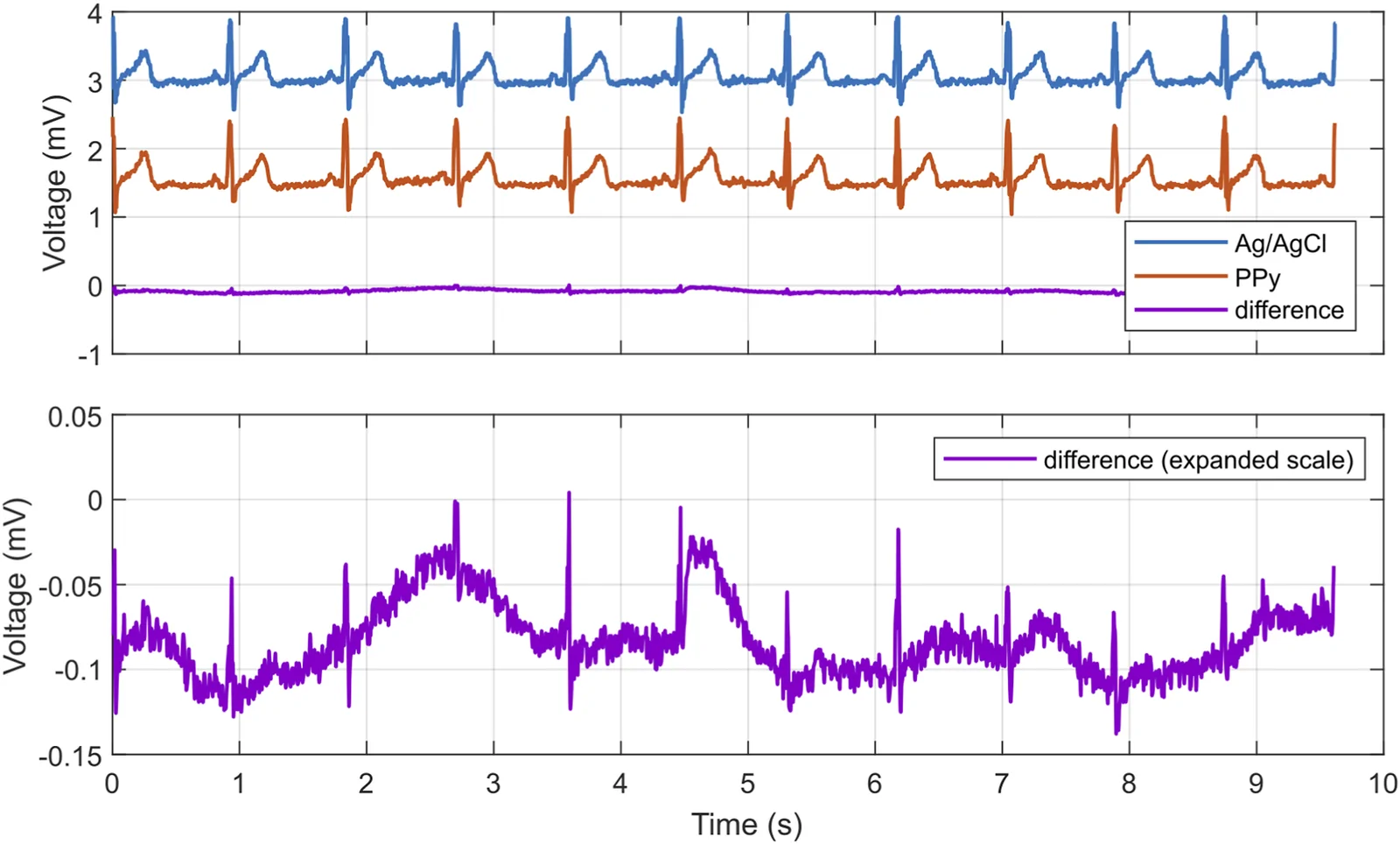
Representative simultaneous bipolar Lead I recordings obtained using reference Ag/AgCl gel electrodes and PPy-coated cotton electrodes in the standard limb configuration. The upper panel shows the Ag/AgCl and PPy signals together with the sample-wise difference waveform ΔECG. For visualization, the Ag/AgCl and PPy traces were vertically shifted by 3.0 and 1.5 mV, respectively; no amplitude scaling was applied. The lower panel shows ΔECG on an expanded voltage scale.

#### 3.1.2 Scenario 2: leadwire transmission fidelity

Following the preliminary material screening described in Section 2.2, three leadwire materials were advanced to quantitative ECG transmission testing against conventional metallic leadwires under unchanged ECG-simulator and acquisition conditions. The experimental procedure and signal-processing methods are described in Sections 2.3.2 and 2.3.5, respectively. Quantitative results are summarized in Table 4.

**TABLE 4:** Quantitative comparison of ECG signal transmission through 1 m candidate leadwire sets relative to conventional metallic leadwires.

Leadwire material	Pearson *r* (−)	MSC_0.5-40_ (−)	*ε*_R_ (%)	ΔSNR (dB)	Δ*σ*_baseline_ (µV)
PANI-coated carbon-fiber bundle	0.9990	0.9813	−1.8	+2.0	+1
Uncoated carbon-fiber bundle	0.9985	0.9598	−2.9	+0.0	−2
PANI-coated technical ribbon	0.9754	0.7619	+0.1	−18.1	+34

Metric definitions and sign conventions are identical to those used in Table 3; detailed definitions are provided in Section 2.3.5.

Pearson correlation coefficients ranged from 0.9754 to 0.9990, while band-mean MSC values ranged from 0.7619 to 0.9813. Among the tested configurations, carbon fiber leadwires coated with PANI exhibited the highest agreement with the metallic reference (*r* = 0.9990, MSC_0.5-40_ = 0.9813, *ε*_R_ = −1.8%) together with a 2.0 dB increase in SNR and a baseline-variability difference of only +1 µV. The uncoated carbon-fiber bundle provided similarly high performance, with (*r* = 0. 9985, MSC_0.5-40_ = 0. 9598, *ε*_R_ = −2.9%).

The PANI-coated technical ribbon exhibited lower spectral coherence (MSC_0.5-40_ = 0.7619), an SNR reduction of 18.1 dB, and an increase in residual baseline variability of 34 µV. The conventional metallic reference exhibited an absolute SNR of approximately 53 dB and baseline variability of 3–5 μV, compared with approximately 35 dB and 30–40 µV for the technical-ribbon configuration.

Figure 12 presents a representative comparison of ECG signals transmitted through the selected 1 m PANI-coated carbon-fiber leadwire set and the conventional metallic reference. The recordings exhibited closely matching waveform morphology. No amplitude scaling was applied, and the two ECG traces were separated by a 1 mV vertical offset for visualization. The lower panel displays the difference waveform ΔECG on an expanded voltage scale.

**FIGURE 12:**
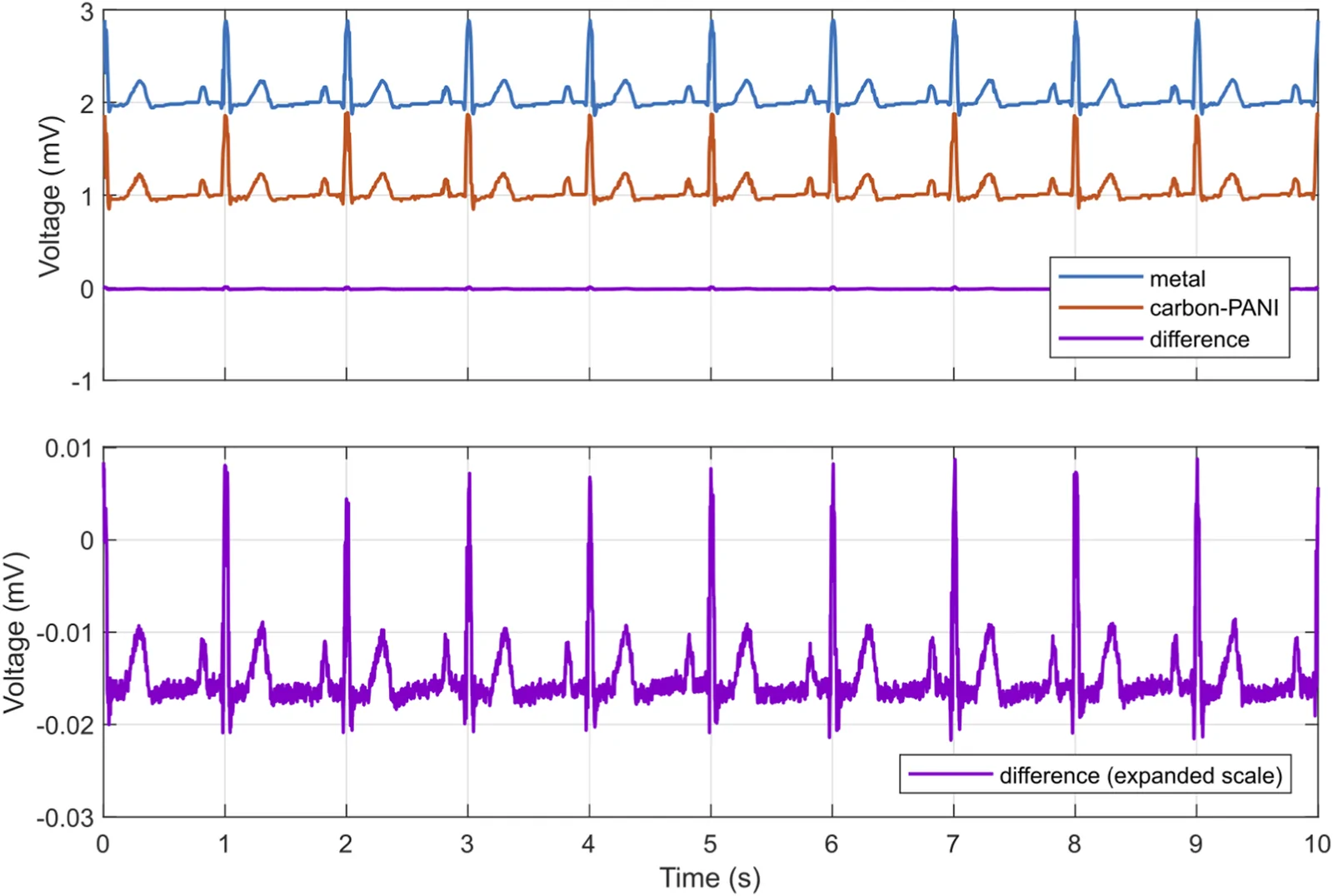
Representative bipolar Einthoven Lead I ECG signals transmitted from the FC12D simulator through conventional metallic leadwires and a 1 m PANI-coated carbon-fiber leadwire set. The upper panel shows the metallic-reference and PANI-coated carbon-fiber recordings together with the difference waveform ΔECG = signal_polymer – signal_metal. For visualization, the two ECG traces were vertically separated by a 1 mV offset; no amplitude scaling was applied. The lower panel shows ΔECG on an expanded voltage scale.

#### 3.1.3 Scenario 3: signal stability of the complete polymer-based electrode–leadwire prototype during CT-table motion

The complete polymer-based electrode–leadwire prototype, comprising PPy-coated cotton electrodes and PANI-coated carbon-fiber leadwires, was evaluated during CT-table translation using the artificial-patient setup. For each bipolar Einthoven lead (I–III), a pre-motion segment and an in-motion segment were selected from the same continuous ECG recording and quantitatively compared. The experimental procedure and signal-processing methods are described in Sections 2.3.3 and 2.3.5, respectively. Quantitative results are summarized in Table 5.

**TABLE 5:** Quantitative comparison of pre-motion and in-motion ECG segments acquired with the complete polymer-based electrode–leadwire prototype during CT-table motion.

Einthoven lead	Pearson *r* ^1^ (−)	RMSE^2^ (mV)	MAE^3^ (mV)	ΔSNR^4^ (dB)	Δ*σ*_baseline_^5^ (µV)
I	0.9511	0.0422	0.0249	−26.0	+41
II	0.9644	0.0401	0.0285	−23.3	+42
III	0.9719	0.0074	0.0053	−24.5	+1

1) *r*–Pearson correlation coefficient. 2) RMSE–Root Mean Square Error. 3) MAE–Mean Absolute Error. 4) ΔSNR–Difference in signal-to-noise ratio between recordings acquired during CT table motion and in the static condition. 5) Δσ_baseline_–Difference in residual baseline variability, quantified as the standard deviation of the pre-QRS isoelectric baseline (80 m window) after bandpass filtering in the ECG frequency range (1–25 Hz).

Positive values of ΔSNR, and Δ*σ*_baseline_ indicate higher signal-to-noise ratio or increased residual baseline variability during CT table motion compared to the static condition, respectively, while negative values indicate a reduction relative to the static reference.

Across the three Einthoven leads, Pearson correlation coefficients between the pre-motion and in-motion segments ranged from 0.9511 to 0.9719. RMSE values ranged from 0.0074 to 0.0422 mV, and MAE values ranged from 0.0053 to 0.0285 mV. Lead III exhibited the highest waveform agreement and the smallest errors (r = 0.9719, RMSE = 0.0074 mV, MAE = 0.0053 mV).

CT-table motion reduced SNR by 23.3–26.0 dB across the three leads. Residual baseline variability increased by 41 and 42 µV in Leads I and II, respectively, whereas only a 1 µV increase was observed in Lead III. Absolute SNR values decreased from approximately 63–66 dB in the pre-motion condition to approximately 37–43 dB during table motion.

### 3.2 Radiological compatibility results

Radiological compatibility was evaluated in two complementary stages: quantitative CT phantom imaging and qualitative veterinary X-ray imaging.

#### 3.2.1 CT phantom radiolucency

The radiolucency of the complete polymer-based electrode–leadwire prototype was evaluated on a HEAD-16 water-equivalent phantom and a custom agar-based phantom and compared directly with a conventional Ag/AgCl electrode–metallic-leadwire system. The polymer-based configurations included PANI-coated carbon-fiber leadwires with and without polyolefin heat-shrink insulation. The experimental setup and CT acquisition and image-evaluation methods are described in Sections 2.3.4 and 2.3.5, respectively. Quantitative visibility results are summarized in Table 6, and the corresponding CT images are presented in Figures 13 and 14.

**TABLE 6:** CT visibility of polymer-based electrode–leadwire components under the evaluated phantom and display conditions.

Phantom	CT view (W/C^1^)	PPy-coated cotton electrode	PANI-coated carbon-fiber leadwire	Velcro backing	Polyolefin leadwire insulation	Ag/AgCl electrode + metallic leadwire
		Visibility |ΔHU|^2^ (HU)	Visibility |ΔHU|^2^ (HU)	Visibility |ΔHU|^2^ (HU)	Visibility |ΔHU|^2^ (HU)	Visibility |ΔHU|^2^ (HU)
HEAD 16	Surview (1500/20)	Invisible n/a	Invisible n/a	Invisible n/a	Invisible n/a	Clearly visible n/a
HEAD 16	Native helical (320/40)	Invisible <40	Invisible <40	Slightly visible ≈80	Slightly visible ≈80	Clearly visible >500
HEAD 16	Native helical, high contrast (1600/–500)	Invisible <40	Invisible <40	Slightly visible ≈80	Slightly visible ≈80	Clearly visible >500
Agar	Surview (1600/–500)	Invisible n/a	Invisible n/a	Invisible n/a	Invisible n/a	Clearly visible n/a
Agar	Native helical, (320/40)	Invisible <40	Invisible <40	Slightly visible ≈80	Slightly visible ≈80	Clearly visible >500
Agar	Native helical, high contrast (1600/–500)	Invisible <40	Invisible <40	Slightly visible ≈80	Slightly visible ≈80	Clearly visible >500

1) W/C denotes window width/window center and specifies the display range used for visual inspection. W/C affects visual conspicuity but does not alter the underlying CT-number values. 2) |ΔHU| represents the absolute difference in mean CT number between the investigated component and the adjacent phantom-background ROI. CT number denotes the X-ray attenuation value of a reconstructed CT voxel expressed in Hounsfield units (HU). Visibility was classified as invisible (<40 HU), slightly visible (40–100 HU), or visible (>100 HU), according to the operational criteria defined in Section 2.3.5. HU-based quantitative evaluation was performed on reconstructed native helical CT images. Surview images were evaluated qualitatively; therefore, HU values are reported as not applicable (n/a). For the native helical images, identical |ΔHU| values apply to both displayed W/C settings because windowing changes visual conspicuity but not the underlying HU values.

**FIGURE 13:**
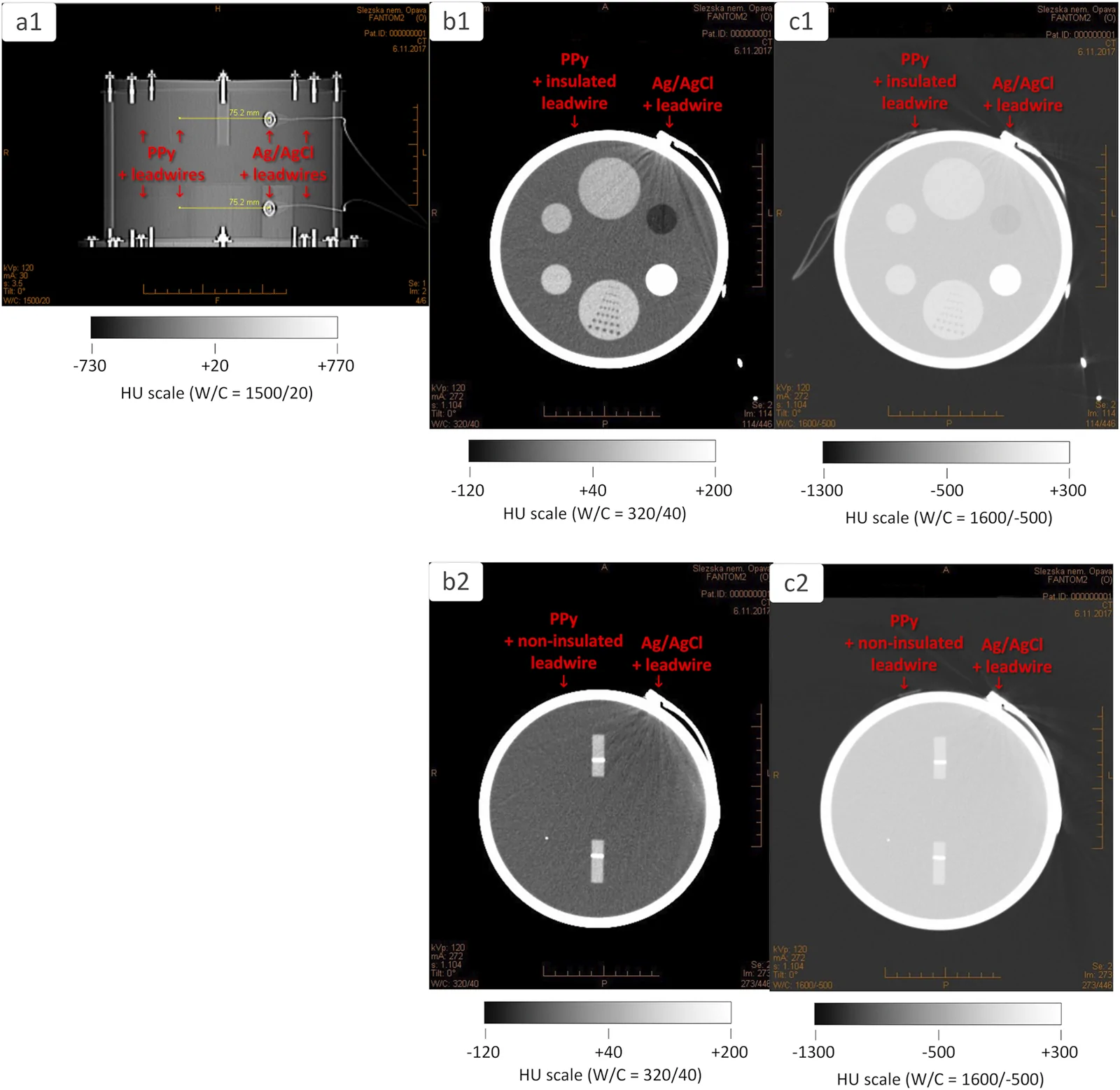
Representative bipolar Einthoven Lead I ECG signals transmitted from the FC12D simulator through conventional metallic leadwires and a 1 m PANI-coated carbon-fiber leadwire set. The upper panel shows the metallic-reference and PANI-coated carbon-fiber recordings together with the difference waveform ΔECG = signal_polymer − signal_metal. For visualization, the two ECG traces were vertically separated by a 1 mV offset; no amplitude scaling was applied. The lower panel shows ΔECG on an expanded voltage scale.

**FIGURE 14:**
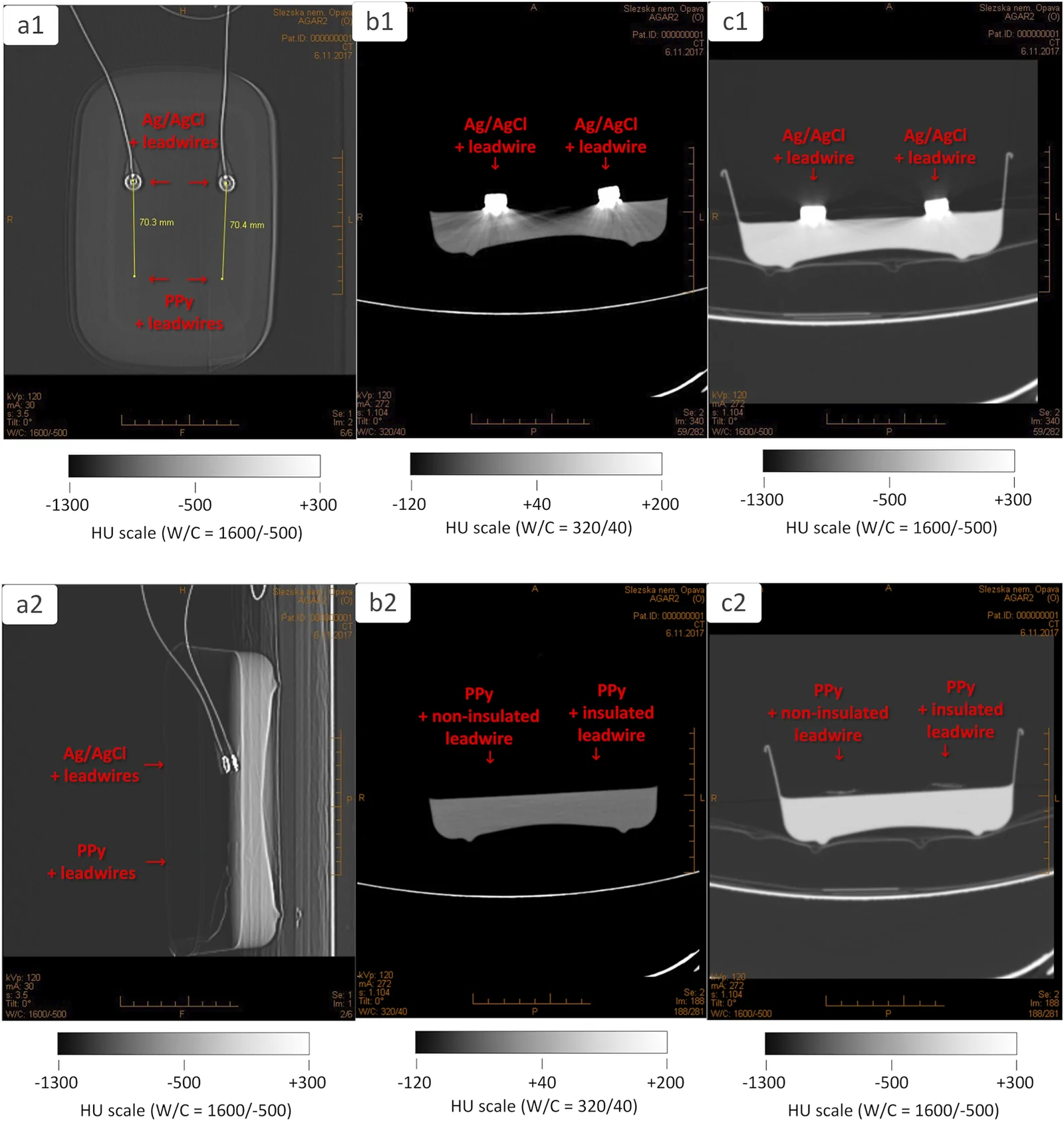
CT Surview and native helical images of the agar-based phantom comparing PPy-coated cotton electrodes connected to PANI-coated carbon-fiber leadwires with and without polyolefin insulation with conventional Ag/AgCl electrodes and metallic leadwires (a1,a2) Surview, W/C = 1600/−500 (b1,b2) native images, W/C = 320/40 (c1,c2) native high-contrast images, W/C = 1600/−500. The 70.3 and 70.4 mm labels indicate the inter-electrode spacing.

Across both phantom models, the PPy-coated electrodes and PANI-coated carbon-fiber leadwires met the operational criterion for invisibility (∣ΔHU∣<40 HU) under all evaluated CT display settings. The Velcro backing and polyolefin insulation were not distinguishable under the default display settings but became slightly visible under high-contrast windowing (W/C = 1600/−500), with ∣ΔHU∣ values of approximately 80 HU.

In contrast, the conventional Ag/AgCl electrodes and metallic leadwires were clearly visible under all evaluated CT display settings, with |ΔHU| values typically exceeding 500 HU, and produced pronounced streak and beam-hardening artifacts. No distinct artifacts originating from the conductive polymer components were observed.

#### 3.2.2 Veterinary X-ray radiolucency

Veterinary X-ray imaging provided a qualitative *in vivo* comparison of the complete polymer-based and conventional metallic electrode–leadwire configurations. The polymer-based configuration comprised PPy-coated cotton electrodes connected to PANI-coated carbon-fiber leadwires insulated with polyolefin heat-shrink tubing. Conventional Ag/AgCl electrodes connected to metallic leadwires served as the reference. Both configurations were positioned simultaneously within the same imaging field. The experimental procedure is described in Section 2.4.2.

Because conventional radiographic imaging does not provide standardized quantitative attenuation values directly comparable with CT-derived Hounsfield units, radiographic visibility was assessed qualitatively based on component conspicuity and the presence of image artifacts.

As shown in Figure 15, the conventional Ag/AgCl electrode and metallic leadwire were clearly visible as high-density radiographic structures and produced pronounced local image obstruction. In contrast, the PPy-coated electrode and PANI-coated carbon-fiber conductor were radiographically inconspicuous. Only a faint outline corresponding to the polyolefin leadwire insulation was discernible, and no distinct radiographic artifact originating from the conductive polymer components was observed.

**FIGURE 15:**
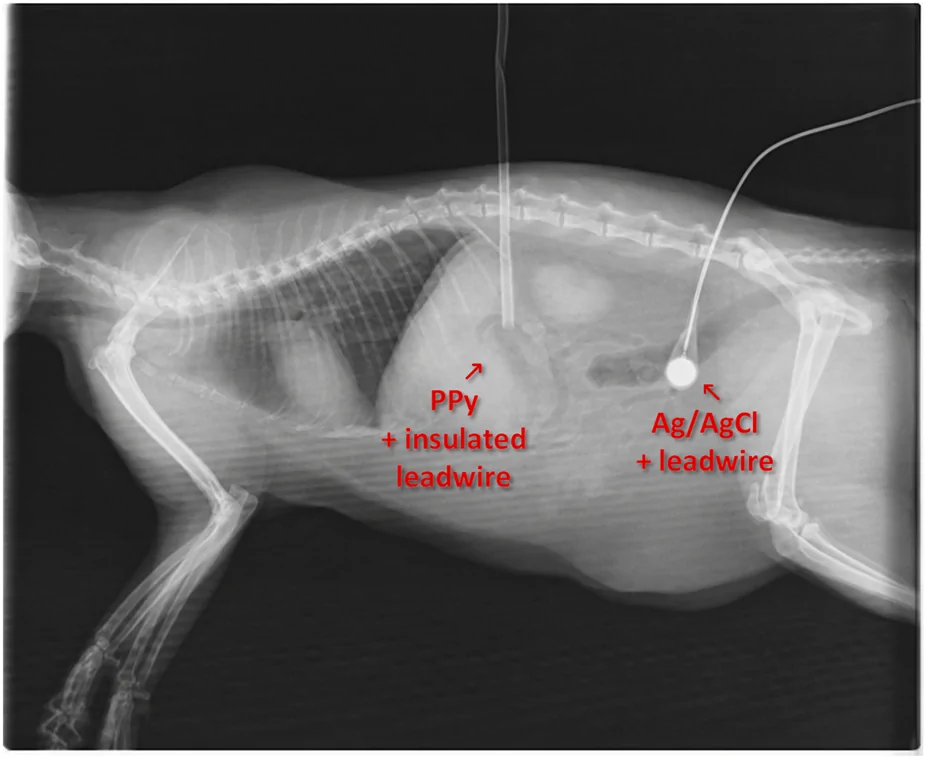
Veterinary radiograph comparing the complete polymer-based and conventional metallic ECG configurations. The polymer-based configuration comprised a PPy-coated cotton electrode connected to an insulated PANI-coated carbon-fiber leadwire, while the reference configuration comprised a conventional Ag/AgCl electrode connected to a metallic leadwire. The conventional components are clearly radiopaque, whereas the conductive polymer components remain radiographically inconspicuous, with only a faint outline corresponding to the polyolefin leadwire insulation.

## 4 Discussion

### 4.1 Principal findings and material performance

The present study demonstrates the feasibility of a fully non-metallic ECG acquisition chain combining PPy-coated cotton electrodes with PANI-coated carbon-fiber leadwires for monitoring during CT and X-ray imaging. Across the standalone electrode measurements, polymer-based recordings showed high agreement with the Ag/AgCl reference, with Pearson correlation coefficients of 0.93–0.99 and magnitude-squared coherence values of up to 0.99. The standard limb configuration generally yielded higher agreement than the Mason–Likar configuration. The larger deviations in the torso placement likely reflect the unavoidable spatial offset between the simultaneously applied polymer and Ag/AgCl electrodes, together with differences in contact pressure and the dry-versus-gel interface. Nevertheless, the PPy-coated cotton electrode maintained high waveform similarity in both configurations and provided the most suitable combination of signal fidelity, mechanical stability, and dry skin contact.

Leadwire testing showed that both uncoated and PANI-coated carbon-fiber bundles preserved ECG waveform morphology with very high fidelity. The PANI-coated 24K carbon-fiber bundle achieved the highest correlation and spectral coherence and exhibited a longitudinal resistance of approximately 11 Ω/m, while retaining flexibility and a fully non-metallic composition. During actual CT-table translation using the artificial-patient setup, all three Einthoven leads maintained high waveform correlation (r > 0.95) and RMSE values not exceeding 0.0422 mV. However, table motion reduced SNR by 23.3–26.0 dB and increased residual baseline variability, particularly in Leads I and II. Thus, ECG morphology remained largely preserved, although dynamic noise increased and clinical diagnostic usability during human CT examinations was not directly validated.

CT and veterinary X-ray imaging confirmed the low radiological conspicuity of the conductive polymer components. In reconstructed CT images, the PPy-coated electrodes and PANI-coated carbon-fiber leadwires remained below the operational visibility criterion of ∣ΔHU∣ < 40 HU, whereas the Velcro backing and polyolefin insulation became only slightly visible under high-contrast windowing. Conventional Ag/AgCl electrodes and metallic leadwires produced pronounced radiographic shadows and CT streak artifacts, while no distinct artifacts originated from the conductive polymer regions. These findings support the potential use of the complete polymer-based electrode–leadwire system during radiological monitoring, although the veterinary X-ray experiment was evaluated qualitatively.

### 4.2 Comparison with recent flexible and radiolucent ECG technologies

The present work extends our previous plant-electrophysiology research on PPy- and PANI-coated substrates (Bajgar et al., 2016) by translating these materials to human surface ECG and integrating a PPy-coated dry textile electrode with a separately developed PANI-coated carbon-fiber leadwire. Following stepwise material screening, the complete passive ECG chain was evaluated at the electrode, leadwire, and system levels, including CT/X-ray imaging and CT-table-motion testing. Table 7 compares the proposed system with representative flexible, conductive-polymer-based, commercial, and radiolucent ECG technologies.

**TABLE 7:** Comparison of the proposed polymer-based electrode–leadwire system with representative flexible, conductive-polymer-based, commercial, and radiolucent ECG technologies.

Study/system	Material and device scope	Representative performance	CT/X-ray compatibility	Main limitation
Commercial reference Ag/AgCl system	Gel electrodes + metallic leadwires	Clinical reference used in this study	Strong imaging artifacts	Gel dependent; metallic components
Flexible ECG electrodes				
Liu et al. (2022)	Flexible AgNW/PVA hydrogel semidry electrode	24-h ECG; SNR >28 dB; stable resistance over 500 cycles	Not evaluated	Electrode only; silver- and hydrogel-based; no radiolucent leadwire
Nan et al. (2025)	Flexible hydrogel wearable ECG system	Low contact impedance; long-term ECG	Not evaluated	Hydrogel and metallic nanomaterials
Kralikova et al. (2023)	Silver-coated dry textile electrode	High ECG similarity to Ag/AgCl	Not evaluated	No radiolucent
Conductive-polymer-based electrodes				
Huang and Lee (2025)	PEDOT:PSS-based textile dry electrode	Surface resistance 2.2 Ω/sq; ECG demonstrated	Not evaluated	Electrode only; no leadwire system
Wang et al. (2025)	Self-adhesive PEDOT:PSS/PVA dry electrode	110 S cm^-1^; 100% elongation; static ECG SNR 28.1 dB; stable 36-h recording	Not evaluated	Electrode only; copper-wire connection; no radiolucent leadwire
Radiolucent ECG systems				
LifeSync commercial radiolucent system	Metal-free radiolucent leadwires and connectors	Commercial ECG performance; quantitative data NR	Radiolucent for fluoroscopy and CT	Gel electrode required; proprietary conductor
Bong et al. (2019)	Graphene/Parylene implantable ECG array	Multichannel ECG demonstrated in a canine model	Graphene electrode sites radiolucent	Implantable array; no radiolucent traces
Present work	PPy-cotton electrode + PANI-carbon-fiber leadwire	108 Ω electrode; 11 Ω/m leadwire; r = 0.93–0.99	CT and X-ray validated	Long-term durability, skin impedance, and *in vivo* CT-motion testing not yet evaluated

As shown in Table 7, many recently reported flexible and polymer-based electrodes are wet, hydrogel-based, or semidry and focus primarily on electrode performance, while repeated reuse, CT/X-ray compatibility, and non-metallic leadwires are generally not evaluated. Radiolucent systems, in contrast, mainly address commercial leadwires or implantable electrode arrays.

The distinguishing contribution of the present work is a complete dry surface-ECG chain combining a PPy-coated cotton electrode with a PANI-coated carbon-fiber leadwire. None of the studies summarized in Table 7, investigated *in-situ* polymerization of carbon-fiber bundles specifically for ECG leadwires or directly compared ECG transmission through uncoated and PANI-coated bundles. Together with CT/X-ray and table-motion testing, this comparison demonstrates the feasibility of a complete radiolucent electrode–leadwire prototype.

### 4.3 Limitations and future work

Several limitations should be acknowledged. First, the CT-motion experiment employed an ECG simulator and a controlled galvanic coupling rather than direct electrode–skin contact. Although this approach enabled isolation of motion-related effects and ensured a reproducible signal source, it does not fully reproduce clinical conditions where perspiration, body hair, respiratory motion, muscle activity, skin deformation, and contact-pressure variations may influence the skin–electrode interface. Human-subject measurements performed in Scenario 1 partially address this aspect; however, future studies should evaluate the proposed system during actual clinical CT examinations.

Second, the present study focused primarily on ECG signal fidelity and radiological compatibility. Long-term durability, repeated cleaning, artificial sweat exposure, cyclic attachment/removal, and extended aging were not systematically investigated. Consequently, while the prototypes remained functional throughout fabrication and experimental testing, the long-term service lifetime and reusability of the system require dedicated future evaluation.

Finally, clinical translation will require larger human-subject studies, optimization of electrode fixation and manufacturing reproducibility, assessment of biocompatibility and cleaning procedures, and appropriate regulatory evaluation. These investigations are necessary to determine whether the demonstrated signal fidelity and radiological compatibility can be maintained under routine clinical conditions.

## 5 Conclusion

This study introduces and validates a novel radiolucent polymer-based biopotential electrode–leadwire prototype for ECG acquisition during X-ray and CT examinations. The prototype consists of polypyrrole (PPy)-coated cotton electrodes and polyaniline (PANI)-coated carbon-fiber leadwires and was specifically designed for use in radiological environments. Through comprehensive testing across ECG signal quality and imaging domains, the proposed fully non-metallic electrode–leadwire prototype demonstrated signal quality comparable to that of standard Ag/AgCl electrodes and metallic leadwire systems and, critically, an absence of clinically relevant radiological artifacts, addressing a long-standing limitation of conventional Ag/AgCl-based ECG monitoring during radiological procedures.

Across three evaluation scenarios, the system preserved ECG waveform morphology and spectral consistency, achieving high Pearson correlation coefficients (>0.95), strong frequency-domain agreement (MSC >0.89), low waveform distortion (RMSE ≤0.05 mV), and diagnostically acceptable signal-to-noise ratios and baseline stability, even under simulated CT table motion.

Radiolucency evaluation on CT phantoms and a veterinary subject confirmed that the conductive polymer components are radiographically inconspicuous, with quantitative HU analysis showing attenuation differences below clinically relevant contrast thresholds. Only auxiliary non-conductive elements (e.g., fixation and insulation) became faintly visible under high-contrast windowing, without generating streaking or beam-hardening artifacts typical of metallic electrodes and leadwires.

Overall, this work demonstrates the feasibility of the proposed polymer-based biopotential electrode–leadwire prototype for dry ECG acquisition during CT and X-ray imaging without electrode removal or repositioning. The dry-electrode, press-on configuration enables rapid application in radiological environments and represents a scalable and clinically relevant alternative with potential for translation into routine diagnostic and interventional workflows.

## Data Availability

The raw data supporting the conclusions of this article will be made available by the authors, without undue reservation.
