# Supplementary material for: Polymer-based biopotential electrode-leadwire prototype for ECG sensing during X-ray and CT examinations
Source: Front Bioeng Biotechnol. 2026 Sep 30;14:1880492. doi: 10.3389/fbioe.2026.1880492 (PMC13662679; doi:10.3389/fbioe.2026.1880492)
Supplement: Supplementary file 1 [file Supplementaryfile1.docx]

# Appendix A - Material Properties of Polymer-Based Electrodes and Leadwires

## A.1 Overview of Investigated Materials

This study evaluated two main conducting polymers – polypyrrole (PPy) and polyaniline (PANI) – chemically polymerized onto various non-metallic substrates to create radiolucent electrodes and leadwires.

- Electrode substrates: cotton fabric, carbon fabric
- Leadwire substrates: polyester thread, linen thread, nylon thread, nylon fishing line, technical ribbon (86% cotton / 14% polyester, 20 mm wide), and carbon-fiber bundles 24K tow

These substrates were selected to meet the application-specific requirements of radiolucency, flexibility, and absence of metallic components, with the aim of minimizing imaging artifacts during simultaneous ECG monitoring and CT or X-ray imaging.

## A.2 Mechanical Properties

Mechanical behavior was evaluated qualitatively during polymerization, sample handling, prototype assembly, repeated bending, and electrode cleaning. The assessment focused on substrate integrity, material cohesion, flexibility, and stability of the deposited polymer layer. For leadwire substrates, particular attention was paid to fraying, stiffness, and structural integrity after polymerization (Table A1):

- Cotton + PPy/PANI: Cotton provided good flexibility and adhesion; PPy-coated cotton showed superior durability compared to PANI-coated variants.
- Carbon fabric: Exhibited excellent conductivity but poor mechanical cohesion, fraying into individual fibers under repeated handling; therefore, excluded from final prototypes.
- Leadwire substrates: Polyester and nylon-maintained stability after polymerization; linen fibers degraded after coating and were unsuitable for prolonged use. Technical ribbon composed of a cotton/polyester blend provided a relatively large surface area and retained sufficient structural integrity after polymerization, although it exhibited moderate stiffness.

Table A1: Summary table of the qualitative mechanical assessment of the investigated substrates.

| **Electrode Material** | **Mechanical properties** |
| --- | --- |
| Cotton coated with PANI, PPy | suitable |
| Carbon fabric | unsuitable |
| **Leadwire Material** |  |
| Carbon fibers  Polyester fiber  Nylon thread  Nylon fishing line  Technical ribbon | suitable |
| Linen fiber | unsuitable |

## A.3 Electrical Properties

Electrical properties of the investigated materials were characterized by direct resistance measurements using an ESCORT 3146A multimeter and point-contact probes. For electrode fabrics, point-to-point resistance R_pp_ was measured at probe separations of 1, 10, and 16 cm. The average of the three measurements was used for a comparative assessment of electrical resistance along the coated textile surface. For leadwire substrates, longitudinal resistance was measured over multiple consecutive sections using probes terminated with crocodile clips and was expressed as resistance per unit length, R_L_ in Ω/m.

- PPy-coated cotton electrodes: mean point-to-point resistance R_pp_ ≈ 108 Ω, indicating low and stable resistance under the applied measurement conditions.
- PANI-coated cotton electrodes: mean point-to-point resistance R_pp_ ≈ 11 kΩ. Although higher than that of the PPy-coated cotton, this resistance was sufficient for ECG acquisition under the tested conditions.
- PANI-coated carbon fiber leadwire: longitudinal resistance R_L_ ≈ 11 Ω/m. This substrate exhibited the lowest measured resistance and was selected for the final leadwire prototypes.
- PANI-coated technical ribbon leadwire: longitudinal resistance R_L_ ≈ 1.6 MΩ/m. ECG transmission was possible, but signal quality was inferior to that obtained with the carbon-fiber leadwire.
- Other polymer-coated leadwires (linen, polyester, nylon): Excessive resistivity; unsuitable for ECG signal transmission.

Although the direct DC resistance values provide a useful comparison of the coated substrates, their relevance to ECG acquisition should be considered together with the R_pp_ substantially larger and frequency-dependent electrode–skin interface impedance. The DC resistance obtained with the multimeter characterizes the conductive textile layer under the specified probe configuration, whereas during ECG acquisition the total impedance also includes the electrode–skin interface. This interface is commonly described by a simplified Randles-type equivalent circuit:

| $Z_{E2}(j\omega)=\frac{R_{2}}{1+j\omega R_{2}C_{2}}+R_{m2}$ | (A1) |
| --- | --- |

where R_2_||C_2_ models the polarization resistance and double-layer capacitance, and R_m2_ accounts for the series resistance of underlying tissue [35]. Typical reported values are R_2_ = 150 kΩ, C_2_ = 17.6 nF and R_m2_ = 5.6 kΩ represent typical values reported. At **DC (0 Hz),** the impedance Z_E2_(0) ≈ 156 kΩ and it decreases with frequency due to the capacitive component ≈ 130 kΩ at 40 Hz. The complete electrode–skin interface impedance depends on skin condition, contact pressure, hydration, electrode geometry, and motion.

The resistance of the dry electrode material acts as an additional series component. For the investigated PANI-coated cotton electrode, the measured point-to-point resistance of approximately 11 kΩ remained below the representative electrode–skin interface impedance and was therefore not expected to dominate the total input impedance. Moreover, because the ECG amplifier used for the simulator measurements had an input resistance of approximately 1 GΩ, the voltage-divider attenuation caused by the electrode and leadwire resistance was small under the tested conditions.

Similarly, even an estimated longitudinal resistance in the megohm-per-meter range for the technical ribbon would produce only limited purely resistive attenuation with a 1 GΩ amplifier input. The inferior signal quality observed for the technical ribbon was therefore more likely attributable to coating inhomogeneity, contact instability, resistance variations along the substrate, and increased susceptibility to interference than to voltage-divider attenuation alone.
